# Molecular basis of CTCF binding polarity in genome folding

**DOI:** 10.1038/s41467-020-19283-x

**Published:** 2020-11-05

**Authors:** Elphège P. Nora, Laura Caccianini, Geoffrey Fudenberg, Kevin So, Vasumathi Kameswaran, Abigail Nagle, Alec Uebersohn, Bassam Hajj, Agnès Le Saux, Antoine Coulon, Leonid A. Mirny, Katherine S. Pollard, Maxime Dahan, Benoit G. Bruneau

**Affiliations:** 1 Gladstone Institutes, San Francisco, CA 94158 USA; 2 Roddenberry Center for Stem Cell Biology and Medicine at Gladstone, San Francisco, CA 94158 USA; 3 Cardiovascular Research Institute, University of California San Francisco, San Francisco, CA 94143 USA; 4 Department of Biochemistry and Biophysics, University of California San Francisco, San Francisco, CA 94143 USA; 5 Laboratoire Physico Chimie Curie, Institut Curie, PSL Research University, Sorbonne Université, CNRS UMR168, 26 Rue D’Ulm, Paris, 75005 France; 6 Institut Curie, PSL Research University, CNRS UMR 3215, INSERM U934, Mammalian Developmental Epigenetics group, F-75005 Paris, France; 7 Sorbonne Université, F-75005 Paris, France; 8 Institut Curie, PSL Research University, Sorbonne Université, CNRS UMR3664, Nuclear Dynamics unit, F-75005 Paris, France; 9 Institute for Medical Engineering and Science and Department of Physics, Massachusetts Institute of Technology, Cambridge, MA 02139 USA; 10 Department of Epidemiology & Biostatistics, Institute for Human Genetics, Quantitative Biology Institute, and Institute for Computational Health Sciences, University of California San Francisco, San Francisco, CA USA; 11 Chan Zuckerberg Biohub, San Francisco, CA USA; 12 Department of Pediatrics, University of California San Francisco, San Francisco, CA 94143 USA; 13 Present Address: University of Washington, Seattle, WA USA; 14 Present Address: University of California Berkeley, Berkeley, CA USA

**Keywords:** Chromatin, Chromosomes

## Abstract

Current models propose that boundaries of mammalian topologically associating domains (TADs) arise from the ability of the CTCF protein to stop extrusion of chromatin loops by cohesin. While the orientation of CTCF motifs determines which pairs of CTCF sites preferentially stabilize loops, the molecular basis of this polarity remains unclear. By combining ChIP-seq and single molecule live imaging we report that CTCF positions cohesin, but does not control its overall binding dynamics on chromatin. Using an inducible complementation system, we find that CTCF mutants lacking the N-terminus cannot insulate TADs properly. Cohesin remains at CTCF sites in this mutant, albeit with reduced enrichment. Given the orientation of CTCF motifs presents the N-terminus towards cohesin as it translocates from the interior of TADs, these observations explain how the orientation of CTCF binding sites translates into genome folding patterns.

## Abstract (web summary)

The boundaries of topologically associating domains (TADs) arise from the ability of the CTCF protein to stop extrusion of chromatin loops by cohesin. Here the authors find that CTCF positions cohesin through its N-terminus but does not control its overall binding dynamics on chromatin, and show how the orientation of CTCF binding sites translates into genome folding patterns.

## Introduction

Mammalian chromosomes are partitioned into topologically associating domains (TADs), which mediate processes ranging from transcriptional regulation to antigen loci recombination^1^. CTCF binding creates TAD boundaries and controls the segmental insulation of chromosome domains^2,3^. The effect of CTCF on chromosome folding is thought to arise from its ability to block loop extrusion by cohesin proteins and modulate their genomic positioning^4–6^. Since the proposal that cohesin could enlarge chromatin loops processively^7^, cohesin complexes have been directly observed extruding DNA loops actively in vitro^8,9^, and found to accumulate at CTCF-binding sites in vivo^10–12^. Intriguingly, cohesin-dependent chromatin loops preferentially engage pairs of CTCF sites with convergent motif orientation^13–15^, and inverting one CTCF motif can lead to repositioning of the corresponding DNA loop^4,16–18^. Biophysical models argue that directional barriers to loop extrusion at CTCF sites are necessary to accurately simulate chromosome folding^4–6^. The molecular basis of this polarity, and of how CTCF constrains cohesin mobility, remains however to be explored.

Here, we investigate the molecular basis for CTCF-binding polarity in genome folding. Combining cohesin ChIP-seq and single-molecule imaging in live cells, we observe that although CTCF localizes cohesin at its binding sites, it does not control overall binding or dynamics of cohesin on chromatin, supporting experimentally that CTCF positions cohesin by blocking its translocation. Using an inducible complementation system, we found that CTCF mutants lacking the N terminus are unable to insulate TADs properly, in spite of normal binding to cognate CTCF sites. Cohesin remained at CTCF sites in this N-terminus mutant, albeit with reduced enrichment. Through systematic truncations of the N terminus, we uncovered several regions important for genome folding and discovered a short protein motif that is both necessary and sufficient to recruit the PDS5A subunit of cohesin in a three-hybrid system. The PDS5A-interacting region of CTCF is distinct from the N-terminal region recently reported to interact with RAD21–SA2 in vitro^19^ and required for cohesin enrichment at CTCF sites^19,20^. This CTCF motif displays homology with the PDS5-binding domain of both WAPL and its competitors SORORIN and HASPIN. Nevertheless, by comparing small mutations within the N terminus, both in isolation and in combination, we show that the recently described RAD21–SA2 interaction domain of CTCF^19^, which also displays homology to WAPL, accounts for most of the functions of the CTCF N terminus in genome folding. Given that the orientation of the CTCF DNA motif presents the CTCF N terminus toward cohesin as it translocates from the interior of TADs, these observations provide a molecular explanation for how the polarity of CTCF-binding sites determines the genomic distribution of chromatin loops.

## Results

### CTCF positions cohesin without controlling its overall binding or dynamics

Two nonexclusive models may account for both localization of cohesin at CTCF sites and directional DNA looping. Cohesin could load at CTCF-binding sites, downstream of the motif, and initiate loop extrusion unidirectionally^21^. Alternatively, cohesin could load throughout TADs and translocate bidirectionally as it extrudes DNA loops, only stopping when it encounters CTCF sites in the proper orientation^4,5^.

**Fig. 1:**
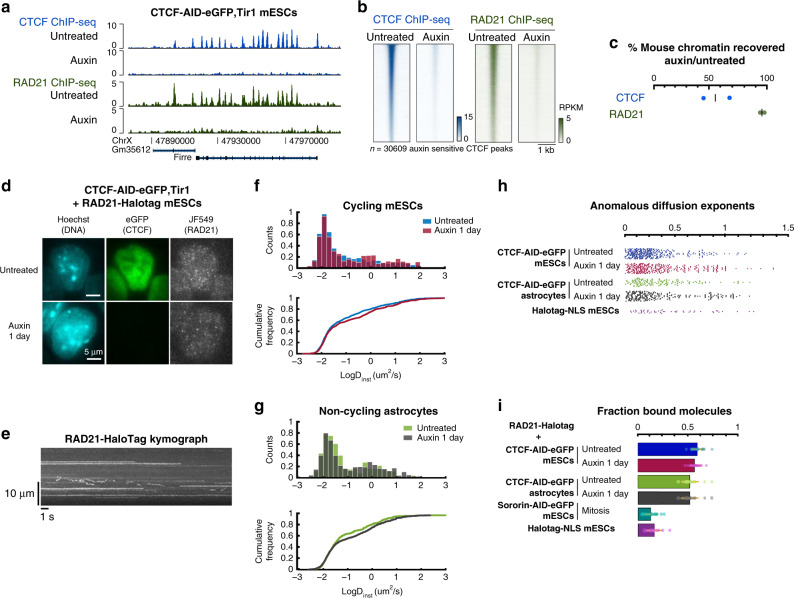
CTCF acts as a positioning but not a loading factor for cohesin. **a**, **b** RAD21 ChIP-seq enrichment at CTCF peaks is lost after depleting CTCF in CTCF–AID mESCs. **c** Percentage of ChIP-seq reads mapping to mouse versus spike-in (*Drosophila*) genomes, using antibodies against either mouse CTCF or mouse RAD21 in CTCF–AID mESCs, normalized to values obtained before CTCF depletion by auxin. Each replicate is plotted separately. **d** HILO imaging of single endogenous cohesin molecules in live CTCF–AID RAD21–HaloTag knock-in mESCs labeled with limiting JF549 ligand (50-ms acquisitions). **e** Part of a kymograph generated by an *xy* line scan across a single cell, illustrating the various diffusion behaviors of RAD21–Halotag in mESCs (50-ms acquisitions). **f** The distribution of diffusion coefficients (*D*_inst_) of RAD21 molecules, although significantly different statistically, is only mildly altered by CTCF depletion. This slight increase in the number of fast-diffusing molecules is observed both in cycling mESCs and **g** noncycling astrocytes. In all, 50-ms acquisitions. Two-sided KS test comparing the cumulative distribution of logD in untreated versus auxin-treated cells: *p* = 0.0196 for mESCs and *p* = 0.0014 for astrocytes, pooling trajectories from all cells. **h** Anomalous diffusion exponents of RAD21 trajectories (50-ms acquisitions) indicating that Rad21 molecules imaged are overwhelmingly subdiffusive (<1). **i** CTCF depletion does not alter the fraction of bound cohesin (5-ms acquisitions). Mitotic block triggered by depleting SORORIN serves as a positive control for free-diffusing cohesin, since most cohesin is unloaded in prophase. Means with standard deviation. See “Methods” for detailed statistics. Source data are provided as a Source Data file.

To test these models, we measured the impact of depleting CTCF on cohesin binding and positioning on chromosomes. As previous studies using inducible CTCF knockout reported that cohesin still displayed ChIP-seq peaks at 80% of initial sites even after 10 days^22^, we sought to achieve more efficient depletion. Using a mouse embryonic stem cell (mESC) line in which CTCF can be degraded by the auxin-inducible degron (AID) system^3^, we observed near-complete disappearance of the cohesin ring subunit RAD21 by ChIP-seq from its initial position at CTCF peaks (Fig. 1a, b). However, spike-in calibration revealed that a RAD21 antibody pulled down an identical amount of chromatin in the absence of CTCF (Fig. 1c). Thus, while cohesin no longer accumulates at CTCF sites in the absence of CTCF, it still associates with chromatin, indicating that it must be redistributed away from CTCF sites—supporting the translocation-and-block model of loop extrusion.

To directly visualize how loss of CTCF may affect cohesin dynamics and association with DNA, we performed single-molecule tracking of RAD21 in WT (Supplementary Fig. 1a–g) and CTCF–AID mESCs (Fig. 1d–g) by targeting both *Rad21* alleles with a HaloTag. As previously reported^23^, 60% of RAD21 molecules were bound to chromatin (Fig. 1i). Depleting CTCF did not affect this fraction, nor the distribution of diffusion coefficients or the anomalous diffusion exponent of RAD21 (Fig. 1f–i). Cell-cycle and sister-chromatid cohesion were not a confounding effect in these imaging modalities (see “Methods”), since we obtained similar results in each single-cycling mESC (Supplementary Fig. 1g), and in noncycling astrocytes (Fig. 1f–g). However, CTCF depletion led to a modest but reproducible increase in the number of quickly diffusing molecules (−1 < Log*D*_inst_ < 0), in both cycling and noncycling cells (Fig. 1f–g). These fast-diffusing molecules were nevertheless not completely free, since they diffused more slowly than unbound cohesin (Log*D*_inst_ > 0), as estimated from imaging cells blocked in early M phase by means of a 6-h depletion of SORORIN (Supplementary Fig. 1h–o). Such a role for CTCF in controlling the diffusion of a small subset of cohesin molecules is in line with recent FRAP experiments, showing that CTCF can stabilize longer-lived RAD21 molecules^19^. Taken together with the spike-in ChIP-seq, our results refute the idea that CTCF promotes bulk loading of cohesin and supports a mechanism whereby CTCF acts by blocking translocating cohesin^24^.

### Systematic evaluation of CTCF domains in chromosome folding

**Fig. 2:**
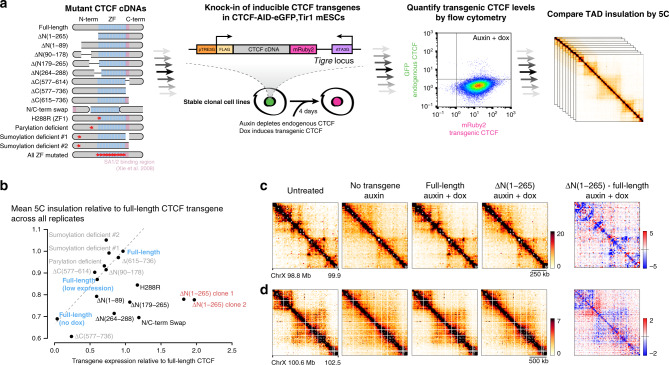
Deletion scanning reveals aminoterminal domains of CTCF that mediate TAD folding. **a** Experimental pipeline for the mutational analysis of CTCF using stable transgenic mESCs. Red stars indicate amino-acid substitutions as detailed in the “Methods”. Flow cytometry confirming homogeneous induction of the transgenes (only full length is shown). Gates were placed using untagged cells. **b** Summary of 44 5C experiments across 16 stable mESC lines treated with dox and auxin. Each data point is the mean of the insulation scores at the six TAD boundaries initially detected in WT-untreated samples, averaged across at least two 5C replicates, and presented as ratios relative to insulation measured in the full-length CTCF transgene. Transgene-expression values correspond to flow-cytometry means across at least two replicates. The dashed line shows the linear regression for the dependence of insulation on transgene expression, and was obtained by comparing cell lines with high, low, or no full-length transgene expression for full-length CTCF. Transgenes with all zinc fingers (ZFs) mutated were poorly expressed and not assessed by 5C. **c**, **d** Snapshots of 5C data binned at 15 kb, and the corresponding differential heatmaps highlighting folding defects in the Δ(1–265) mutants. Units are normalized counts—see “Methods”.

We next investigated how CTCF mediates TAD insulation. Mutational analysis of CTCF is challenging because CTCF is essential for long-term cell survival^3,21^, and mutations altering CTCF protein stability or CTCF binding will de facto alter cohesin positioning and TAD folding—since insulation of TADs relates quantitatively to CTCF levels^3^. To overcome these obstacles, we used a complementation system where inducible CTCF cDNA transgenes are stably targeted in CTCF–AID cells, so that auxin degrades endogenous CTCF and doxycycline triggers expression of the CTCF transgene (Fig. 2a). Precise comparison of expression levels between cell lines was achieved by flow cytometry for mRuby2, fused in-frame to transgenic CTCF. TAD folding was surveyed across all genotypes by chromosome-conformation capture carbon copy (5C) using a previously validated design^3^. To calibrate our assay, we analyzed two independent lines expressing the full-length CTCF cDNA at either a high or low level, together with one cell line not expressing the transgene. Insulation (“Methods”) scaled linearly with transgene expression (Fig. 2b, dashed line). Expression of the full-length transgene (high) was approximately one-fifth of endogenous CTCF–AID-eGFP, which is less than half untagged CTCF (Supplementary Fig. 3a–c).

We first deleted C(577–614), which contains a region expected to mediate the interaction between CTCF and cohesin based on in vitro data^25^, and encompasses the C-terminal internal RNA-binding region, RBR_i_ (Supplementary Figs. 2d and 3a)^26,27^. ΔC(577–614) is expressed at around 60% of the level of the full-length transgene, confirming that the region contributes to CTCF stability (Supplementary Fig. 3b)^27^. ΔC(577–614) displayed lower DNA binding by ChIP-seq (Supplementary Fig. 3e–g) and rescued insulation as expected based on its expression level (Fig. 2b and Supplementary Fig. 3c, d). Furthermore, ΔC(577–614) co-immunoprecipitated with the cohesin subunit SA2 from nuclear extracts (Supplementary Fig. 3h), in line with other studies^26,27^. C(577–614) is therefore dispensable for connecting CTCF and cohesin in vivo, and appears to contribute minimally to TAD folding beyond promoting CTCF binding (it is possible that our 5C assay did not detect subtle changes at the subset of micro-C peaks recently reported to change in this mutant)^27^. Another domain must therefore mediate cohesin blocking and overall directional loop retention by CTCF.

### CTCF N(1–265) mediates chromatin folding into TADs

We proceeded to establish an additional 12 stable cell lines, each harboring a different mutated CTCF cDNA, leaving the core of the DNA-binding domain intact (central zinc-finger (ZF) array—Fig. 2a and Supplementary Fig. 2d). Several CTCF mutants failed to rescue TAD insulation to the extent expected from their expression levels (Fig. 2b). Deletion of the entire N-terminal domain ΔN(1–265) had the most impact (Fig. 2c, d). Within the N terminus, multiple subregions participate to the ability of CTCF to insulate TADs (Fig. 2b): ΔN(1–89) triggered a mild but detectable insulation defect, while ΔN(179–265) had a more pronounced effect. ΔN(264–288), which overlaps one RNA-binding region and ZF1, as well as mutation of the ZF1 itself (H288R), also led to insulation defects and is characterized further in a parallel study^28^.

As for the C-terminal domain, while the single ΔC(577–736) clone analyzed affected insulation, expression of the mutant protein was very low. Given that two other tiling deletions ΔC(577–614) and ΔC(615–736) expressed at higher levels did not disrupt TAD insulation noticeably, we conclude that the N terminus is the most potent domain of CTCF for insulating TADs.

### CTCF N(1–265) participates in retaining cohesin at CTCF sites

**Fig. 3:**
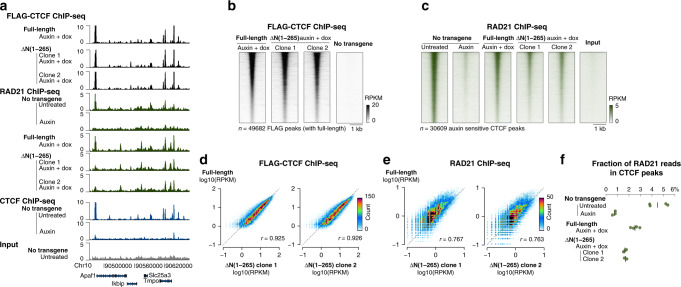
The CTCF N terminus participates in but is not strictly required for cohesin positioning at CTCF sites. **a** ChIP-seq track snapshot, **b**, **c** density plots, **d**, **e** scatterplots, and **f** fraction of reads in peak (FRIP) scores indicates that RAD21 is still detected at CTCF peaks^3^ in cells expressing CTCF ΔN(1–265), albeit with a twofold reduced enrichment compared to the full-length CTCF transgene. CTCF ChIP-seq data were obtained from ref. ^3^.

To understand the pronounced chromatin-folding defects in ΔN(1–265), we measured binding of transgenic CTCF and endogenous Rad21 by ChIP-seq. Deleting the entire N terminus did not alter CTCF binding, as indicated by FLAG pulldown (Fig. 3). RAD21 enrichment at FLAG–CTCF peaks remained detectable in the ΔN(1–265) mutant, but was reduced twofold (Fig. 3). Therefore, proper retention of cohesin at CTCF sites requires N(1–265), indicating that the CTCF N terminus either participates in inhibiting cohesin translocation (thereby promoting insulation) or—nonexclusively—protects blocked cohesin from unloading (thereby bolstering 5C peaks between CTCF sites). These observations are in line with a parallel study concluding that the N terminus is required for RAD21 occupancy at CTCF sites^20^.

Given that deleting the CTCF N terminus led to milder insulation defects than complete CTCF depletion, and that deleting the C terminus had little-to-no effect, the ZF array mediates some degree of insulation and must therefore participate in halting cohesin translocation. The ZF domain confers to CTCF an unusually long residence time for a transcription factor^23,29^, as well as uniquely distorts DNA^30^ and positions nucleosomes^31^ in a fashion that might interfere with loop extrusion by cohesin.

### CTCF N(13–33) can recruit PDS5A via a motif shared with WAPL and SORORIN

**Fig. 4:**
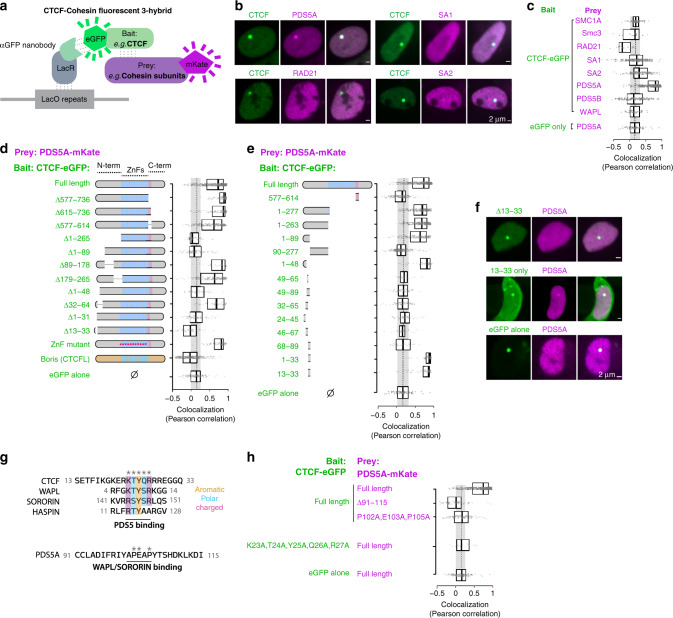
The CTCF N terminus can interact with the PDS5A cohesin subunit via a motif shared with WAPL and SORORIN. **a** Fluorescent three-hybrid setup testing the ability of CTCF to recruit cohesin subunits in BHK-LacO cells. **b**, **c** PDS5A is the only subunit recruited by CTCF by F3H. Each data point corresponds to the Pearson correlation between the green (CTCF) and red (cohesin subunits) channels at one GFP-positive LacO array. High values denote high colocalization of CTCF and cohesin subunits at the array. Boxplots indicate the first and third quartile and median. **d** Deleting CTCF N(13–33) prevents PDS5A recruitment in F3H. **e**, **f** N(13–33)-eGFP is sufficient to recruit PDS5A by F3H. **g** CTCF N(23–27) aligns with the known PDS5-binding region of WAPL, SORORIN, and HASPIN reported to interact with the APEAP motif of PDS5. **h** Mutation of the APEAP motif of PDS5A prevents its recruitment by CTCF in the F3H assay. Alanine substitution of CTCF N(23–27) prevents PDS5A recruitment by CTCF.

Our results suggested that N(1–265) may contain one region (possibly more given Fig. 2b) able to interact directly or indirectly with cohesin and alter its behavior during loop extrusion. To test this hypothesis, we tethered CTCF to a LacO array (or the nuclear periphery, Supplementary Fig. 4) and monitored the recruitment of transiently overexpressed cohesin subunits by fluorescent three-hybrid (F3H)^32^ (Fig. 4a). The only cohesin subunit recruited by CTCF in this assay was PDS5A (Fig. 4b, c). ΔN(1–265) completely abrogated PDS5A recruitment, as did the smaller ΔN(13–33) (Fig. 4d). Conversely, fusing CTCF N(13–33) to eGFP was sufficient to elicit PDS5A recruitment (Fig. 4e).

Sequence alignment revealed that CTCF N(13–33) contains a KTYQR motif highly analogous to the known PDS5-binding domains of WAPL, SORORIN, and HASPIN (Fig. 4g)^33,34^. Alanine substitution of CTCF KTYQR abrogated PDS5A recruitment by F3H. Reciprocally, alanine substitution of the APEAP motif in PDS5, known to bind WAPL and SORORIN, also abrogated its recruitment by CTCF in F3H (Fig. 4h). Altogether, this indicates that CTCF binds the same region in PDS5 as SORORIN and WAPL. This is especially interesting, given that SORORIN binding to PDS5 through this region is known to shield PDS5 from the releasing activity of WAPL, thereby opposing cohesin unloading^35^. Our observations raise the possibility that CTCF might act similarly.

It remains unclear at this stage why CTCF cannot recruit PDS5B in F3H, in spite of the region around the APEAP motif being highly similar between PDS5A and PDS5B. Human and mouse CTCF 13–33 are 100% identical (Supplementary Fig. 4), with extremely high conservation throughout the protein, including the N terminus, up to fishes. Supporting our observations with mouse orthologs, we observed that human CTCF also recruits human PDS5A, and much more efficiently than human PDS5B (Supplementary Fig. 4e). It is possible that a remote segment unique to PDS5B interferes with its recruitment by CTCF.

Given that PDS5 regulates cohesin dynamics^2^ and opposes translocation^36–38^, these findings prompted us to explore further how CTCF can block cohesin and stabilize DNA loops. First, because binding of PDS5 and NIPBL to the cohesin ring is mutually exclusive^36^, CTCF may prevent NIPBL from promoting ATP hydrolysis and cohesin translocation, thereby blocking cohesin at CTCF sites. Second, CTCF may interfere with completion of the unloading process, employing its N terminus to disconnect PDS5A from the cohesin unloader WAPL. These observations also offer insight as to why depleting PDS5A and PDS5B diminishes Hi–C peaks between CTCF sites^2^.

In the context of loop extrusion, competition between NIPBL and PDS5 would regulate cohesin step rate or velocity, and the affinity of CTCF for PDS5 would tune that step rate to zero locally, at CTCF sites, thereby instructing insulation in a site-specific manner. Supporting the notion that PDS5 can indeed dampen loop-extrusion velocity independently of unloading, loss of PDS5 triggers global chromosome condensation without augmenting cohesin residence time as dramatically as loss of WAPL^2^. Furthermore, PDS5 depletion prevents the appearance of ectopic Hi–C peaks observed upon loss of WAPL^2^, indicating that PDS5 acts upstream of WAPL at CTCF sites. More generally, locally tuning cohesin-extrusion dynamics may be employed by additional transcription factors other than CTCF^39^, and we envision that it will prove a general principle with consequences on long-range transcriptional regulation.

### CTCF N(226–228) mediates most but not all effects of CTCF in TAD folding

Importantly, the CTCF–PDS5A axis mediated by N(13–33) cannot account for all functions of the N(1–265) region in TAD folding, since ΔN(1–89) exhibits only partial insulation defects (Fig. 2b). Other N-terminal regions we identified by 5C, such as N(179–265) and possibly the N-terminal RNA-binding region around ZF1, may also participate in functionally connecting CTCF and cohesin by means that are not readily captured by the F3H assay. Although we were able to reproducibly co-immunoprecipitate all CTCF truncations with an anti-SA1 antibody (Supplementary Fig. 3h), detection of the interaction was very sensitive to extraction conditions. Furthermore, we have been unable to detect cohesin proteins after the reciprocal pulldown of CTCF. These co-immunoprecipitation data might not reflect a robust and stable interaction between CTCF and cohesin. Our observations are concordant with a parallel study demonstrating that several regions in the CTCF N terminus mediate cohesin retention, and that the CTCF N terminus is necessary for Hi–C peaks between TAD boundaries^20^.

**Fig. 5:**
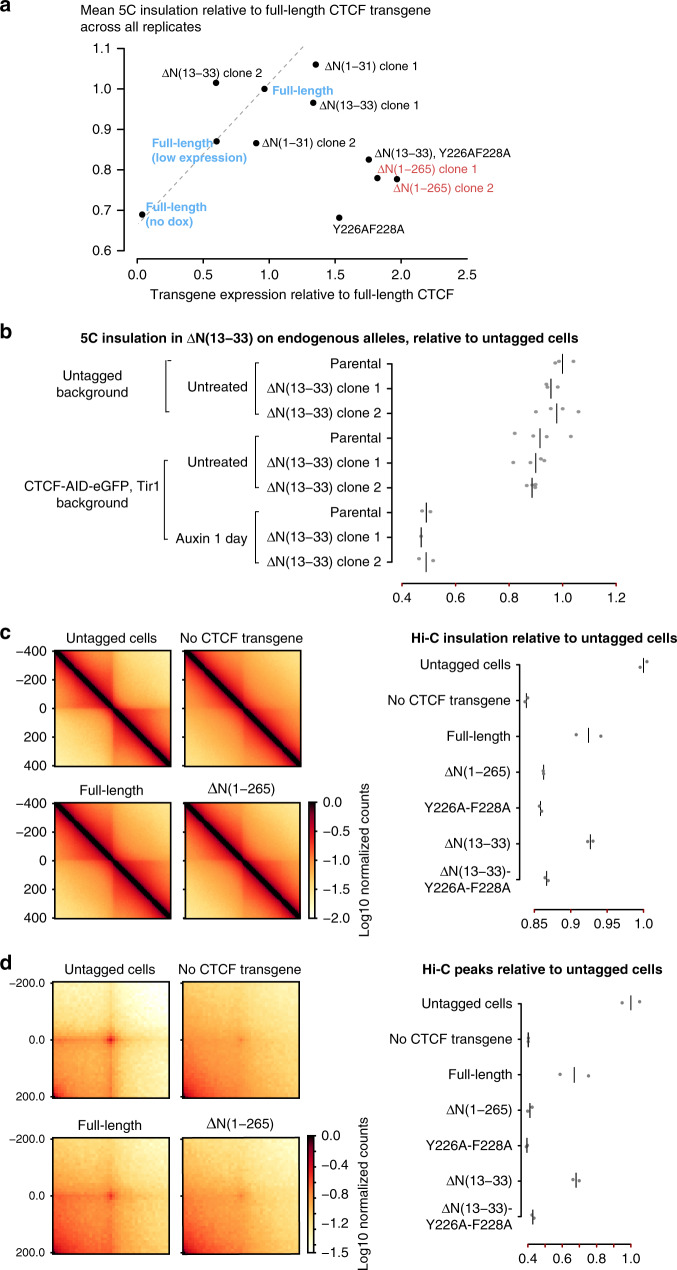
5C and Hi–C analysis of N-terminal mutations of CTCF. **a** Summary of 5C experiments in stable mESC lines harboring CTCF transgenes with N-terminal mutations and treated with dox and auxin as in Fig. 2b. Colored data points are reproduced from Fig. 2b for comparison. **b** TAD insulation analysis from 5C data obtained on cells with region N(13–33) from endogenous CTCF alleles. Each point is the average insulation measured in one 5C replicate and notch marks the median. **c** Hi–C in stable mESC lines expressing CTCF transgenes. Aggregate insulation scores are depicted next to aggregate heatmaps of select genotypes. Each point is the average insulation measured in one Hi–C replicate and notch marks the average. **d** Same data as in **b** for aggregate peak analysis.

Three out of four clonal cell lines expressing either ΔN(1–33) or ΔN(13–33) rescue CTCF transgenes that exhibited slight insulation defects by 5C (Fig. 5a). The effects were mild, however, indicating that ΔN(13–33) does not mediate the effects of the entire ΔN(1–265) N terminus. In order to circumvent possible complications from analyzing transgenes, we deleted N(13–33) homozygously from the endogenous *Ctcf* alleles, in both untagged and CTCF–AID-eGFP,Tir1 cells. Endogenous ΔN(1–33) did not trigger obvious growth defects, and chromosome folding was very mildly affected by 5C (Fig. 5b), across 4 replicates of 4 clonal cell lines.

The CTCF N terminus was recently discovered to bind RAD21–SA2 in vitro via amino acids N(226–230), and the Y226A/F228A mutation triggers almost complete loss of Hi–C peaks between TAD boundaries^19^. Given that N(226–230) can compete out a WAPL-binding site on RAD21–SA2 in vitro^19^, we explored whether N(226–230) might compensate the deletion of the N(13–33) region, which we show also has the potential to compete out WAPL binding (this time to PDS5A, Fig. 4). We therefore leveraged our inducible rescue system to mutate N(13–33) either alone or in combination with N(226–230). We also assessed the impact of these N-terminal mutations relative to either deletion of the entire N terminus or complete loss of CTCF, using both 5C (Fig. 5a) and Hi–C (Fig. 5c, d).

Meta-analyses of TAD insulation and loops genome-wide by Hi–C, using boundaries previously identified by ultra-deep sequencing^40^, enabled us to use shallow sequencing across seven genotypes in replicate (Fig. 5c, d, Supplementary Fig. 5). In line with our 5C data, deleting N(13–33) did not reduce insulation or Hi–C peak strength, even in combination with Y226A/F228A. We conclude that the PDS5A-interacting domain of CTCF is dispensable for chromosome folding as monitored by our assays. It remains possible that the CTCF–PDS5A interaction is relevant for pathways we have not assayed. Of note, Y226A/F228A alone exhibited similar Hi–C defects as the entire N-terminal deletion, which itself retained more insulation than full CTCF depletion—consistent with our 5C analyses shown in Fig. 2. We conclude that the N(226–228) region is the most potent domain of CTCF in genome folding. Future experiments will address whether the other disruptive N-terminal truncations detected in Fig. 2b and other studies^20,28,41,42^ alter the function of this domain.

## Discussion

**Fig. 6:**
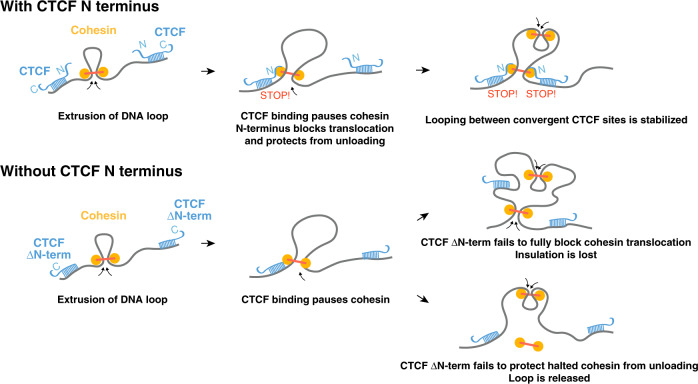
Summary model for the role of the CTCF N terminus in chromosome folding. Upon encountering a bound CTCF site, cohesin halts, irrespective of motif orientation^18,72^. Because of the nonpalindromic nature of the CTCF DNA motif, the effect of the CTCF N terminus on cohesin retention and DNA loop stabilization is polarized to one side of CTCF-binding site. Altogether, these events result in pairs of interacting TAD boundaries being preferentially populated by CTCF motifs in convergent orientation. Upon deleting the N terminus of CTCF, cohesin occupancy is diminished but still detectable, indicating that cohesin still pauses upon encountering bound CTCF sites. Loss of cohesin occupancy may reflect either or both decreased ability of truncated CTCF to block cohesin (leading to insulation defects) and decreased ability of truncated CTCF to protect halted cohesin from unloading (leading to loss of the DNA loop). See Supplementary Fig. 6.

Altogether, our data reveal the importance of the N-terminus portion of the CTCF protein in stabilizing cohesin at CTCF-binding sites, providing a molecular explanation for how CTCF-binding site polarity instructs chromosome folding (Fig. 6 and Supplementary Fig. 6). The inducible degron-based genetic complementation approach presented here allowed comparing the effect of either mutating or acutely depleting CTCF, in a context where endogenous CTCF is not present. Our conclusions are in line with two recent studies that used distinct strategies to ascertain the importance of the N terminus for cohesin retention and genome folding. Li et al.^19^ introduced a point mutation at the endogenous locus (Y226A/F228A) without comparing to full depletion of CTCF, and Pugacheva et al.^20^ complemented a cell line where CTCF binding is disabled at a subset of sites interspersed between unaffected sites.

The importance of the CTCF N terminus draws support from evolutionary data: while the ZF domain of CTCF is highly conserved across bilateria^43^, vertebrate and invertebrate N termini are highly divergent overall. In *Drosophila*, CTCF-binding sites also overlap cohesin ChIP-seq peaks^44^ (Supplementary Fig. 7), but do not exhibit motif orientation bias at domain borders^45^ and do not anchor Hi–C peaks^46,47^. This reinforces the notion that, while the conserved ZF domain is an impediment to cohesin translocation, the mammalian N terminus is required to fully retain cohesin and stabilize chromatin loops as they appear by Hi–C. While the CTCF N terminus is highly conserved across mammals, it is highly divergent from that of its paralog BORIS/CTCFL. BORIS does not interact with PDS5A (Fig. 4d), lacks homology to the RAD21–SA1-interaction domain in CTCF, and does not share the functions of CTCF in genome architecture^20,48–50^.

Altogether, our observations also explain why TAD boundaries are preferentially populated by pairs of CTCF sites with binding sites in a convergent orientation, and why inverting a CTCF site impairs chromatin interactions, in spite of leaving cohesin ChIP enrichment unchanged^18^. Indeed, orientation of the CTCF motif ensures that cohesin translocating from the inner portion of TADs encounters the N terminus of CTCF (Fig. 6 and Supplementary Fig. 6). When the N terminus is placed C-terminally of the ZF array, CTCF is unable to rescue TAD folding, indicating that oriented presentation of the N terminus is crucial (Fig. 2b). Finally, our observations also provide insight as to why depleting WAPL triggers accumulation of DNA loops between nonconvergent CTCF sites^2,51^: the unloading complex is necessary to release loops held by cohesin at CTCF sites, even when cohesin halts by encountering the C-terminal pole of CTCF- binding site (Supplementary Fig. 4). This would account for the cohesin traffic jam at CTCF motifs in divergent orientation in cells devoid of WAPL^52^. In summary, our results point toward additional functions of CTCF beyond cohesin blocking, namely protecting from unloading, and pave the way for further mechanistic dissection of the process.

## Methods

### Cell culture

Parental WT mESC E14Tg2a (karyotype 19, XY, 129/Ola isogenic background) and subclones were cultured in DMEM + Glutamax (ThermoFisher cat 10566-016) supplemented with 15% fetal bovine serum (ThermoFisher SH30071.03), 550 µM b-mercaptoethanol (ThermoFisher 21985023), 1 mM sodium pyruvate (ThermoFisher 11360-070), 1× nonessential amino acids (ThermoFisher 11140-50), and 10^4^ U of Leukemia-inhibitory factor (Millipore ESG1107). Cells were maintained at a density of 0.2–1.5 × 10^5^ cells/cm^2^ by passaging using TrypLE (12563011) every 24–48 h on 0.1% gelatin-coated dishes (Millipore cat ES-006-B) at 37 °C and 7% CO_2_. The medium was changed daily when cells were not passaged. Cells were checked for mycoplasma infection every 3–4 months and tested negative. The CTCF-AID mESCs (full genotype CTCF-AID-eGFP, Tir1(random insertion)) were described as cell line #EN52.9.1 in ref. ^3^. A full list of the cell lines used and generated in this study, with unique identifier numbers, can be found in Supplementary Table 1.

To establish neural progenitors and astrocytes, CTCF–AID mESCs were seeded at around 0.1 million cells in a 75-cm^2^ gelatinized dish in mESC medium. The following day, cells were rinsed twice in 1× phosphate-buffered saline and switched to N2B27 medium (50% DMEM/F12 medium: Gibco 31330-038, 50% Neurobasal medium: Gibco 21103-049, 1× Glutamax Gibco 35050061, 0.5× B27 Gibco 17504-044, 1× N2 Millipore SCM012, and 0.1 mM 2-mercaptoethanol) (ThermoFisher 21985023) and changed daily. After 7 days, cells were detached using TryplE and seeded on nongelatinized bacterial dishes for suspension culture at 3 million cells per 75 cm^2^ and cultured in N2B27 containing 10 ng/mL EGF and FGF (Peprotech 315-09 and 100-18B). After 3 days, floating aggregates were seeded on gelatinized dishes. After 2–4 days, cells were dissociated using Accutase and passaged twice on gelatinized dishes in N2B27 + EGF + FGF and cryopreserved after expansion. For differentiation into quiescent astrocytes, adherent NPC cultures were washed twice with N2B27 and cultured for at least 48 h with N2B27 + 10 ng/mL BMP4 (R&D Systems 314-BP-010).

Schneider’s Drosophila Line 2 (S2) cells were obtained from ATCC and cultured in Schneider’s Drosophila Medium (ThermoFisher 21720001) with 10% heat-inactivated FBS (ThermoFisher SH30071.03) at 28 °C according to the ThermoFisher protocol.

The Baby Hamster Kidney (BHK) LacO clone #2 used for Fluorescent three-hybrid was created in the laboratory of David Spector^53^ and kindly provided by Pierre-Antoine Desfossez.

AID depletion was triggered using 500 mM of Indole-3-acetic acid sodium salt (auxin analog) (Sigma-Aldrich Cat #I5148) final, diluted in culture medium. TetO promoters were induced using 1 μg/ml doxycycline final, diluted in culture medium. Single-molecule imaging in CTCF–AID cells was performed after 1 day of auxin treatment, to minimize secondary effects. ChIP-seq and Hi–C was performed after 2 days of auxin (+dox) treatment to enable comparison with previous ChIP-seq and Hi–C data^3^. 5C was performed after 4 days of auxin (+dox) treatment, where the effect of CTCF depletion and the difference with the CTCF full-length transgene rescue were maximal^3^.

### Plasmid construction

Plasmids were assembled using Gibson assembly (SBI MC010B-1) or restriction ligation. Mouse cDNAs were used for CTCF and cohesin transgenes, and cloned after by reverse transcription of mESC (E14Tg2a) mRNAs (SuperscriptIII, ThermoFisher). BORIS/CTCFL cDNA was synthesized as a gblock by IDT. Human cDNAs were produced from WTC11 hiPSCs. The GFP nanobody–LacR fusion plasmid^32^ was kindly provided by Heinrich Leonhardt and Cristina Cardoso. Targeting vectors driving doxycycline-inducible CTCF cDNAs were assembled by modifying the pEN366 vector^3^ (Addgene 156432).

Parylation-deficient CTCF was created by alanine substitution of the eight glutamic acid residues between positions 215 and 244, known to obliterate parylation^54^. The N-terminal sumoylation site was obliterated by introducing the previously described^55^ K75R mutation.

CTCF amino-acid number refers to UniProtKB—Q61164 (CTCF_MOUSE).

The list of plasmids generated in this study can found in the Supplementary information. Key plasmids and annotated sequence maps are available through Addgene (https://www.addgene.org/Elphege_Nora/).

### Genome engineering

For transfection, plasmids were prepared using the Nucleobond Maxi kit (Macherey Nagel) followed by isopropanol precipitation. Constructs were not linearized.

To knock in TetO-CTCF cDNAs at the *Tigre* locus, CTCF–AID, Tir1(random insertion) clone EN52.9.1^3^ was transfected using using the Neon system (Thermofisher) using a 100-µL tip with 1 million cells at 1400 V, 10 ms, and 3 pulses. Five micrograms of the Cas9-*Tigre* sgRNA vector pX330-EN1201^3^ (Addgene #92144) and 15 µg of the targeting construct were used. The CTCF transgenes encode a puromycin-selection cassette under the PGK promoter, flanked by FRT sites. After electroporation, cells were seeded in a 9-cm^2^ well and left to recover for 48 h. Cells were plated at limited dilution and grown for around 7 days in the presence of puromycin at 1 µg/mL, until single colonies could be picked. Individual clones were genotyped by polymerase chain reaction (PCR) and analyzed by flow cytometry for induction of the CTCF-mRuby2 transgene on a MACSQuant analyzer. Homozygous clones were identified by PCR, and those driving expression as close as possible as the control cells harboring the full-length CTCF transgene were expanded and cryopreserved. See Supplementary information.

To knock in the Halotag at RAD21, mESCs (E14Tg2a or CTCF–AID, Tir1(random)) were transfected using the Neon system (Thermofisher) using a 100-µL tip with 1 million cells at 1400 V, 10 ms, and 3 pulses. Five micrograms of the Cas9 sgRNA vector pX330–EN1082 (see Supplementary information) and 15 µg of targeting construct pEN313 were used (see Supplementary information). We sought to shorten the isolation of homozygous clones with the selection cassette removed. To avoid two rounds of subcloning, we adopted the following strategy. After electroporation, cells were seeded in a 9-cm^2^ well and left to recover for 48 h. Geneticin was then added to the media at 200 µg/mL without subcloning: cells were selected as a heterogeneous pool of homozygous and heterozygous cells for around 10 days, at which stage over 70% of the cells showed nuclear fluorescence after addition of the fluorescent Halotag ligand. This heterogeneous pool of cells was then used for transfection with the Neon system using a 10-µL tip and 0.1 million cells with 250 ng of a flippase-expressing plasmid (pCAGGS-FlpO-IRES-puro)^56^ in order to trigger FRT recombination and excision of the blasticidin-selection cassette. After electroporation, cells were seeded in a 9-cm^2^ well and left to recover for 48 h, then subcloned by transferring into a 78-cm^2^ petri dish from which two serial 1:10 dilutions were seeded in an additional two dishes. After 7–8 days of culture without antibiotic selection, single colonies were manually picked, transferred into a 96-well plate, dissociated, and replated. Clones were then genotyped by PCR for homozygous insertion of the Halotag, checked for geneticin sensitivity, expanded, and cryopreserved.

We noticed that the RAD21–Halotag cells derived from the CTCF–AID, Tir1(random), clone EN52.9.1, stopped responding to auxin upon differentiation. We therefore used RAD21–Halotag, introduced Tir1 at the Tigre locus using pX330-EN1201 (Addgene #92144) and pEN396 vectors (Addgene #92142), and isolated a homozygous knock-in clone that we used to introduce an AID-eGFP cassette at both endogenous alleles of CTCF using pEN244 (Addgene #92144) and (pCAGGS-FlpO-IRES-puro)^56^. We noticed that when targeted at *Tigre*, Tir1 expression remained stable upon differentiation.

To create Sororin-AID cells, RAD21–Halotag cells were transfected using the Neon system (Thermofisher) using a 100-µL tip with 1 million cells at 1400 V, 10 ms, and 3 pulses with 5 µg of the Cas9 sgRNA vector pX330-EN1680 (see Supplementary information) and 15 µg of the targeting construct pEN487 (see Supplementary information). A homozygous clone was isolated, used for co-transfection with (pCAGGS-FlpO-IRES-puro)^56^ to remove the blasticidin-selection cassette. Tir1 was then introduced at rosa26 using vectors pX330-EN479 (Addgene #86234) and pEN114 (Addgene # 92143). Homozygous clones were identified by PCR.

To delete the nucleotides encoding for CTCF(13–33) from the endogenous allele, we created a targeting vector consisting of 1 kb upstream and downstream of the region to delete clones into pUC19 (Bruneaulab vector pEN715). We co-transfected this plasmid together with the sgRNA vector pX459–EN2328 (derived from pX459, Cas9-2A-puro, Addgene #62988—see Supplementary information). We used the Neon system (Thermofisher) using a 100-µL tip with 1 million cells at 1400 V, 10 ms, and 3 pulses with 15 µg of pEN715 and 5 µg of pX459–EN2328. One day later, puromycin was added at 1 µg/mL. One day later, cells were split for limiting dilution in 10-cm plates with puromycin. Starting 1 day later, the medium was changed daily without puromycin. Single colonies were picked manually and genotyped by PCR. Deletions were also confirmed from cDNA generated from the selected clones, and that no WT CTCF cDNA was produced by these cells. These mutant cells did not exhibit noticeable growth defects.

The list of cell lines generated in this study and the corresponding CRISPR sgRNAs can found in Supplementary Data 1.

### ChIP-seq

Preparation of spike-in chromatin from S2 cells—cells were detached from culture dishes by splashing them gently but thoroughly with culture medium, and transfered to a 15-mL conical tube before spinning at 1000*g* for 3 min. Cells were resuspended at 10^6^ cells/mL in complete S2 culture medium at room temperature. In total, 270 µL of 37% Formaldehyde (Electron Microscopy Sciences) was taken for a final concentration of 1%, and agitated on an orbital shaker for 10 min @ RT. In total, 510 µL of 2.5 M glycine (final concentration 125 mM) was added, and cells were left agitating for 5 min @ RT, then spun at 1000*g* for 2 min, 4 C. Fixed cells were washed once in 1 mL of cold 1×PBS–0.125 M glycine, and spun at 1000*g* for 3 min, 4 C. Cells were used for sonication without prior freezing, as we noticed that snap freezing dramatically altered shearing efficiency. Fresh cell pellets were resuspended in 1 mL of Cell lysis buffer (20 mM Tris HCl, pH 8.0, 85 mM KCl, 0.5% IGEPAL, and 1× Halt protease inhibitors, ThermoFisher PI78425) and incubated on ice for 10 min. Nuclei were pelleted by spinning at 2500*g* for 5 min at 4 C and lysed in 50 mM Tris HCl, pH 8.0, 10 mM EDTA, 1% sodium dodecyl sulfate, and 1× Halt protease inhibitors for 30 min on ice. Chromatin was sheared using a Covaris S220 ultrasonicator 5% Duty cycle, 5 intensity, and 200 cycles/burst for 7 min. Debris were pelleted by centrigugation at 1500*g* for 5 min. The supernatent was transferred into a new tube, and glycerol was added at 10% final concentration before freezing at −80 °C as single-use aliquots. For each ChIP experiment, 600 ng of *Drosophila* chromatin (as estimated from the amount of DNA retrieved after reverse cross-linking) was used in combination with sonicated chromatin obtained from 10 million mESCs.

RAD21 ChIP-seq in Fig. 1—The first set of RAD21 ChIP-seq was performed in parallel of CTCF ChIP-seq in the CTCF–AID mESC clone EN52.9.1 published in 2017^3^, using 10 mg of antibody Abcam ab992 together with 40 ng of *Drosophila melanogaster* spike-in chromatin (Active motif 53083) and spike-in antibody (Active motif 61686). These tracks are tagged as “2017protocol” in Supplementary Data 2 and companion GEO submission of this study.

RAD21 and FLAG ChIP-seq in Fig. 3—FLAG and RAD21 ChIP-seq in mESCs containing CTCF rescue transgenes, as well as replicates of the parental CTCF–AID line EN52.9.1 post 2017, were prepared with a protocol differing from data in Fig. 1 by the lysis and wash buffers. For the full-length transgene, we used the high-expressing clone (EN133.10) to be closest to the expression level of the ΔN(1–265) clones.

For fixation, mESCs were dissociated using TrypLE and resuspended in 10% FBS in PBS, counted, and adjusted to 1 million cells per mL. Formaldehyde was then added to 1% final concentration followed by 10 min of incubation at room temperature. Quenching was performed by adding 2.5 M glycine–PBS to 0.125 M final concentration followed by 5 min of incubation at room temperature, 15 min of incubation at 4 °C, and centrifugation at 200*g* for 5 min at 4 °C, resuspended with 0.125 M glycine in PBS at 10 million cells per mL, aliquoted, spun at 200*g* for 5 min at 4 °C, and snap-frozen on dry ice.

Fixed cells were thawed on ice, resuspended in ice-cold 20 mM Tris HCl, pH 8.0, 85 mM KCl, 0.5% IGEPAL and 1× HALT protease inhibitor, counted and readjusted to obtain 10 million cells in total exactly, incubated on ice for 15 min, centrifuged at 500*g* for 5 min at 4 °C, resuspended in 1 mL of 20 mM Tris HCl, pH 8.0, 0.1% SDS, 0.5% sodium deoxycholate, and 1× HALT protease inhibitor, and transferred to a MilliTube (Covaris). Chromatin was sheared on a Covaris S2 sonicator for 15 cycles at 5% duty cycle, intensity 8, 200 cycles per burst in a waterbath maintained at 4 °C, using 1 min of sonication—30 s of rest, resulting in fragments. Samples were clarified by centrifugation at 18,000*g* at 4 °C for 10 min. Supernatants were transferred to 15-mL conical tubes, and 600 ng of spike-in *Drosophila* chromatin (home made) was added. A 10% of the mixture was saved as input and the rest was diluted to 5 mL with ice-cold 16.7 mM Tris Hcl, pH 7.4, 167 mM NaCl, 0.01% SDS, 1.1% Triton X-100, 1.2 mM EDTA, and 1× protease inhibitor. In total, 10 μg of anti-FLAG (Millipore-Sigma F1804) or anti-RAD21 (Abcam 992) together with 4 μg of spike-in antibody (anti-H2Av, Active motif) was added alongside with 40 μL of prewashed protein G Dynabeads (ThermoFisher) followed by overnight incubation at 4 °C on a rotator. Beads were collected using a magnetic stand, transferred into 2-mL tubes, and washed with 1 mL twice for 5 min with 20 mM Tris HCl, pH 8.0, 150 mM NaCl, 2 mM EDTA, 0.1% SDS, and 1% Triton X-100, twice for 5 min with 20 mM Tris HCl, pH 8.0, 500 mM NaCl, 2 mM EDTA, 0.1% SDS, and 1% Triton X-100, and twice for 5 min with 10 mM Tris HCl, pH 8.0, 0.25 M LiCl, 1 mM EDTA, 1% NP40, and 1% sodium deoxycholate, and rinsed twice with 1× TE buffer. DNA was eluted twice by resuspending washed beads with 50 µL of 1% SDS, 0.1 M NaHCO_3_, and incubating for 30 min and pooling eluates. Saved input DNA was diluted in the same buffer and treated similarly. Of 10 mg/ml, 1 ml of DNAse-free RNAse A was added, and eluates were incubated at 37 °C for 30 min, prior to addition of 1 µl of 20 mg/ml Proteinase K and 12 µl of 5 M NaCl, and overnight incubation at 65 °C. The next day, DNA was cleaned either using Ampure Beads (FLAG ChIPs) or Qiagen PCR cleanup minelute kit, eluting in 32 mL. DNA was then used for library preparation exactly as described^3^, using the entire eluate for ChIP-seq and 40 ng for inputs.

### ChIP-seq analysis

Mapping and peak calling were performed as exactly as described previously^3^ using mm9 assembly: Fastq files were trimmed using the fastq-mcf program, aligned to the mm9 reference genome with the bowtie2 software^57^. Reads with a mapq score of 30 or greater were retained, using Samtools. Heatmap visualization and integration with RNA-seq was performed using the Easeq version 1.03 software^58^. Published^3^ CTCF ChIP-seq peaks in untreated and auxin-treated CTCF–AID mESCs were used to identify total and auxin-sensitive CTCF peaks. The fraction of reads in peak scores were calculated by the proportion of uniquely mapping reads within auxin-sensitive CTCF peaks compared to the total number of uniquely mapping reads, and excluding genomic regions known to display artificial ChIP-seq signal^59^ retrieved from https://sites.google.com/site/anshulkundaje/projects/blacklists.

The RAD21 ChIP-seq presented in Fig. 1, and identified as Rad21_(2017_protocol) in Supplementary Data 2, was generated in parallel of the CTCF ChIP-seq data published^3^ in GEO series GSE98671. We used matching inputs for the analysis as those were generated in parallel (see Supplementary Data 2). These samples were generated using the commercial Active motif spike-in reagents (spike-in chromatin cat#52083 and spike-in antibody cat#61686), where spike-in calibration yielded consistent results.

For RAD21 ChIP-seq in mESCs with the CTCF transgenes (Fig. 3), we noticed that spike-in normalization gave inconsistent results, artificially rescaling up or down RAD21 scores beyond reason and inconsistently between replicates. While these samples were generated using homemade *Drosophila* chromatin from S2 cells (ATCC cat# CRL-1963) and Active motif spike-in antibody cat#61686, we observed similar inconsistency using commercial *Drosophila* spike-in chromatin from Active motif cat#52083 (not shown). To avoid normalization artifacts, we display FLAG and RAD21 analyses without recalibration. Reads were mapped separately to mm9 and dm3 as described^3^, eliminating low-quality reads, PCR duplicates, and multimapping reads. Tracks and density plots were generated using Easeq^58^ http://easeq.net/.

The list of FLAG (CTCF) peaks from cells expressing the full-length FLAG–CTCF, used in Fig. 3, is provided as Supplementary information in the GEO series of this paper. It corresponds to the overlapping peaks from libraries ENC178 and ENC205 (Supplementary Data 2), excluding blacklisted genomic intervals.

For mapping *Drosophila* RAD21 enrichment at CTCF sites in Kc167 cells, published^44^ datasets from accession GSE63518 were mapped to dm3, and peak calling was performed as exactly as described previously^3^.

### Chromosome-conformation capture carbon copy (5C)

5C was performed exactly as described^3^ with the same 5C oligonucleotide pool, which corresponds to a single alternating design of 486 Forward and 504 Reverse oligos, spanning 4.5 Mb across mm9 chrX:98837477–103425147. Note that all cells used here are XY with a single active X chromosome.

### 5C analysis

Sequencing and mapping were performed as described^3^ using mm9 reference coordinates. Matrices were then iteratively corrected at the fragment level and normalized to sum to 1e6. Iterative correction was performed on raw unbinned matrices (fragment level from the alternating 5C primer design) using iterative_correction_asymmteric with default values (cooltools, https://github.com/open2c/cooltools). 5C heatmap data depicted in the figures were obtained after binning the corrected matrices at 15 kb by taking the median over all primer pairs that fall within each pair of bins.

To minimize possible artifacts when calculating insulation scores, we binned the matrices at 20 kb by taking the mean over all primer pairs that fall within each pair of bins. The first two diagonals of the binned matrix were then filled with the mean of the second diagonal. Combined insulation scores for each sample were calculated for the binned corrected matrices by aggregating over the same set of boundary positions across samples. Boundaries were identified in untreated CTCF–AID mESCs without any CTCF transgene (GEO accession GSE98671 samples GSM2609248, GSM2609253, and GSM2609256)^3^ by taking the minima of the insulation profile, as described previously^3^. Insulation scores were calculated with a 100-kb window, as described previously^3^. These minima were then filtered to exclude those that are shared with those upon auxin-mediated degradation of CTCF–AID for 4 days in mESCs (GSM2609254 and GSM2609259) (to eliminate CTCF-independent boundaries—e.g., compartment transitions). Combined insulation scores averaged across all replicates (Fig. 2) were calculated as the mean across boundary positions and averaged across replicas, for each cell line separately. To calculate insulation relative to full-length transgenes, averages of mutant cDNAs were divided by the average obtained with the reference full-length transgene. The genomic positions of the CTCF-dependent boundaries used were boundary1 chrX:99151148–99171148, boundary2 chrX:99411148–99431148, boundary3 chrX:100451148–100471148, boundary4 chrX:100671148–100691148, boundary5 chrX:101211148–101231148, and boundary6 chrX:103211148–103231148.

Similar results were obtained when using the four most visually prominent boundaries. Differential heatmaps were generated by binning each matrix independently and subtracting the 5C counts from the reference matrix.

### Hi–C sample preparation

Hi–C was performed with the Arima Genomics kit following the manufacturer’s recommendations and using 1 million cells per reaction.

### Hi–C analysis

We processed each Hi–C dataset using *distiller* (https://github.com/open2c/distiller)^60^, mapping reads to mm10 and saving processed data in the *cooler* format (https://github.com/open2c/cooler^61^) at 10-kb resolution. For each library, around 70% of initial reads were valid Hi–C pairs with >90% in *cis*. We used iterative correction^62^ to remove biases using cooler balance (filters: mad_max=8, min_count=20).

Aggregate boundary analyses used 4753 boundaries called on Bonev et al.^40^ 10-kb binned ESC data. Data by Bonev et al. were also mapped using *distiller* to mm10 and iteratively corrected. Boundaries were called using *calculate_insulation_score* with a 200-kb window, and with additional stringent thresholds boundary_strength >0.25, log2_insulation_score <0. Average boundary maps for individual datasets were constructed using *CoolerSnipper* from *cooltools* (https://github.com/open2c/cooltools)^63^ to collect and aggregate ±400-kb regions around each boundary, and then normalized to the average value of the second diagonal. To quantify boundary strength for a dataset, we took the ratio between map values for positions spanning the boundary to those shifted to be completely upstream or downstream of the boundary, as performed in earlier studies^64^. Here, we used positions separated by up to 80 kb and shifted the areas by 80 kb upstream and downstream. Aggregate peak analyses used 4450 dots (Hi–C loops) called on Bonev et al.^40^ 10-kb binned ESC data, processed as above. Dots were called using *cooltools* dotcaller with default parameters. Average peak maps were constructed using *CoolerSnipper* from *cooltools* to aggregate ±200 kb paired regions around each set of peak anchors, and then normalized to the average value of 200 kb inward from both anchors. To calculate the peak score for a replicate fold enrichment at the average peak, we computed the ratio between the value of the central pixel with the average of the values in a 6 × 6-pixel region shifted 10 pixels down and to the left, as described in previous studies^13^.

### Live single-molecule imaging

Microscopy setup—Single-molecule imaging was performed on an epifluorescence-inverted microscope (IX71, Olympus) in HILO illumination^65^. In all, a 500-mm achromatic lens conjugates the slit to the specimen plane to achieve a proper HILO. The lens focuses the excitation beam on the back-focal plane of a 150× objective lens (UApo N 150× TIRF 1.45 NA, O.I., Olympus, France). The lens is mounted on a translation stage together with a metallic mirror that sends the beam to the microscope. Displacement of the translation stage allows a precise positioning of the focused beam at the back-focal plane of the objective without influencing the lens-BFP distance. Thanks to this configuration, it is possible to adjust the tilting of the laser beam at the output of the objective and thus the effective thickness of the tilted light-sheet excitation at the specimen.

Efficient separation between the excitation and emission was achieved with a fluorescence cube containing a quad-band dichroic mirror (FF409/493/573/652-Di02-25 × 36, SEMROCK) together with adequate emission filters. The setup is provided with a 561-nm laser (Sapphire 561, Coherent, Santa Clara, CA, USA), a 488-nm laser (488LM-200, ERROL, France), and a 405-nm laser (405LM-200, ERROL, France). Lasers were tuned via an acousto-optical tunable filter (AOTFnC-400-650-TN, A&A Optoelectronic, France) and controlled by a homemade interface in Micromanager v1.4.20^66^. The signal was acquired with an EM-CCD camera (iXonEM DV860DCS-BV, Andor, Ireland) run in frame-transfer mode.

Acquisitions—To perform single-molecule-tracking experiments, cells (both mESC and astrocytes) were grown on circular petri dishes with glass bottom (MatTek, Part No: P35G-1.5-14-C) coated with fibronectin (Millipore SAS cat# FC010-5mg). Cells were seeded at a density of 3 × 10^5^/cm^2^ the day before the experiments, in culture medium based on Fluorobrite DMEM for mESCs (ThermoFisher A1896701) and in phenol-red N2B27 with BMP4 for astrocytes (ThermoFisher 12348017). We underline the importance of performing single-molecule imaging in phenol-red free medium to both reduce the background fluorescence and minimize localization errors.

The experiments were performed 20 h (labeled as 1 day) after adding auxin to culture medium. To achieve single-molecule labeling, cells were incubated with 1 pM of Halo-JF549 for 20 min at room temperature (incubation followed by a first rinsing step, 15-min wait, and another rinsing). While waiting for the second rinsing step, cells were incubated with 1 μM Hoechst and consequently washed to minimize the fluorophores unbound in solution. All washings were performed using cell- culture medium; the coverslips treated with auxin were washed with medium enriched with auxin. During the experiments, cells were kept at 37 °C and 5% CO_2_ with a Tokai Hit heating system (INUBG2E-PPZI).

To locate nuclei, cells were stained with Hoechst 33342 (bisBenzimide H 33342 trihydrochloride, Sigma-Aldrich, ref 14533), excited with 405-nm light. The CTCF-GFP was imaged in the 488-nm channel. To track Cohesin-Halo-JF549, the sample was excited with the 561-nm laser. At least 5000 frames were recorded in a continuous imaging regime, the laser being controlled by the camera. Laser power was approximately 0.1 kW/cm^2^ and adjusted depending on the exposure time in order to keep the amount of excitation photons constant.

To determine the fraction of bound molecules, we acquired images in a continuous regime at a frame rate of 197 Hz (5 ms). For the analysis of the dynamics (MSD) and the residence time, we acquired videos at a rate of 20 Hz (50 ms).

Quantification of photobleaching—To characterize the photobleaching of the organic dye used for our single-particle-tracking experiments (SPT), we acquired movies in the same imaging conditions of the SPT experiments in terms of laser power and exposure. Cells were stained with the JF549 organic dye^67^ at 1 nM for bulk labeling. The plot in *Extended* Fig. 1 shows the average normalized bleaching curve for acquisitions made with an exposure time of 50 ms with the same laser power used for the SPT experiments.

### Analysis of single-particle-tracking data

To localize the single emitters and build the trajectories, we used SLIMfast^68^, implemented in Matlab and based on the MTT algorithm^69^. The point spread function of a single emitter is fitted with a 2D gaussian, whose center corresponds to the position of the fluorophore with a subpixel resolution.

Analysis of bound fractions—To quantify the fraction of bound molecules, we used data acquired at 5-ms exposure in a continuous imaging regime. The actual frame-rate acquisition is 197 Hz (5.08 ms), due to the frame-transfer lag to the camera. We chose to use the data from the fastest acquisition rate to include the fastest-diffusing population, which blurred when imaging with 50 ms of exposure time.

Particles were tracked as described above, and we computed the distribution of the step sizes of the protein of interest. The trajectories consisted of at least one step, or two localizations. A two-state model was chosen to fit our data. The computation of the fraction of bound molecules is corrected for the subset of free molecules that may leave the focal plane^70^. The fit was performed on the cumulative distribution function to avoid biases due to the binning choice.

Residence times—To further characterize the binding kinetics, we extrapolated the trajectories that stayed confined in a circular area of radius *r* = 2 pixels for the whole duration. With this pool of “immobile” trajectories, we built the distribution of residence times and consequently computed the Survival Probability. Such distribution of residence times is defined as the inverse cumulative probability, or the probability for a molecule to have a life longer than t_0_: $\mathop {\smallint }\limits_{t_0}^\infty P(t)dt$.

Given the intrinsic limitations of single-molecule imaging when probing very stable binding events (as for cohesin), we use the Survival Probability curves to qualitatively sample the discrepancies between the different biological conditions.

Analysis of diffusion dynamics—The trajectories obtained from experiments at 50 ms were analyzed with custom codes implemented in Matlab. First, we computed the time-averaged mean- squared displacement (MSD) as MSD = 〈*xt* + *n*∆*t* − *xt*〉, where *x*(*t*) is the position at time point *t*, *n* = 1, 2 …, *N*, with *N* = maximum number of time points in a trajectory, and 〈〉 indicating the ensemble average over all the possible time lags of one individual trajectory.

We selected the trajectories with at least ten localizations. In spite of the low JF549 ligand concentration, the beginning of the videos is very dense in point emitters. We therefore cut the first hundred frames of the raw movies, and we only performed tracking on images with approximately ten molecules per frame. We did not threshold data used to quantify the fraction of bound molecules nor to the estimation of the Survival Probability.

Once computed the MSD, we extrapolated what we call the instantaneous diffusion coefficient (*D*_inst_) from each trajectory by fitting the MSD from point 2 to point 6. We followed the common approach of performing a linear fit, assuming a purely Brownian motion at the beginning of the MSD^68,71^.

Detailed statistics—See Supplementary Data 3 for the number of trajectories analyzed in each condition. For auxin-treated Sororin cells blocked in mitosis, we only performed 5 ms of acquisition because >80% of molecules are freely diffusing (Fig. 1), resulting in blurred signal when acquiring for 50 ms. Statistics related to *Extended* Fig. 1g: see Supplementary Data 3.

### Immunostaining

mESCs were grown on glass coverslips, fixed with 3% formaldehyde in 1× PBS for 10′ at room temperature. Permeabilization was carried out in 0.5% Triton followed by blocking with 1% bovine serum albumin diluted in 1× PBS (Gemini cat 700-110) for 15 min at room temperature. Primary antibody incubation was performed at room temperature for 45 min (Monoclonal ANTI-FLAG^®^ M2 antibody produced in mouse Millipore-Sigma F1804 at 1/250 dilution), followed by three 5-min washes in 1× PBS, secondary antibody incubation (AlexaFluor594 Goat anti-Mouse IgG Invitrogen A-11005 at 1/10,000 dilution), three 5-min washes in 1× PBS, counterstaining with DAPI, and mounting in 90% glycerol—0.1× PBS—0.1% p-phenylenediamine, pH 9. Images were acquired on a Zeiss spinning disk with 60× objective. In order to avoid loss of loosely attaching mitotic cells for the H3S10 immunostaining in Sororin-AID cells, cells were detached with TryplE, spun in culture medium, resuspended in PBS, and let to attach for 10 min in 1× PBS 25-μl droplets spotted onto 0.1% poly-l-lysine-coated coverslips. Cells were then processed as described above, except that the primary antibody used was Anti-H3S10Ph, rabbit polyclonal, Millipore 05-636.

### Fluorescent three-hybrid

BHK-LacO clone #2 (previously described^32,53^) was seeded in eight-well ibidi slides (cat. 80826) 16,000 cells per chamber. After about 24 h, the medium was changed, and transfection was carried out using lipofectamine 2000, with 150 ng of GFP nanobody–LacR, 150 ng of GFP plasmid, and 300 ng of mKate2 plasmid (Lipofectamine 3000 gave lower transfection efficiency). After 24 h, cells were washed once with 1× PBS and incubated for 10 min with 1× PBS containing 3% formaldehyde (Electron Microscopy Sciences), then rinsed three times with 1× PBS, incubated with 0.5% Triton X in 1× PBS for 5 min and 1 µg/ml DAPI, rinsed twice with 1× PBS, and left in 1× PBS for imaging. Typically, 20–40% of cells displayed green fluorescence at the LacO array.

Images were acquired as 3D stacks on a Zeiss spinning-disk microscope using 405-, 488-, and 561-nm excitation lasers with a 60× oil objective. Images were analyzed in imageJ with the JACoP plugin to calculate the Pearson correlation between red and green channels within a 12 × 12 × 8 X × Y × Z box manually placed on each GFP-positive LacO array. As recommended in the original F3H protocol^32^, cells that did not receive both plasmids were excluded by filtering out cells with low signal intensity in the red channel. Using different thresholds did not affect the conclusions. For the boxplots presented in Fig. 4 and extended Fig. 4, we used a threshold of 5000 for the red channel (and no threshold for the green channel), in reference to the data in the Source Data file. Boxplots show the results measured over at least 30 LacO arrays across at least two independent transfections carried on different days, typically.

### Flow cytometry

mESCs were dissociated with TryplE, resuspended in culture medium, spun, and resuspended in 4% FBS–PBS before live flow cytometry on a MACSQuant instrument (Miltenyibiotec). Dissociation, wash, and flow buffers were supplemented with auxin, when appropriate, to avoid re-expression of the CTCF–AID–eGFP fusion. Analysis was performed using the Flowjo sowftware.

### Western blots

mESCs were dissociated, resuspended in culture medium, pelleted, washed in PBS, pelleted again, and kept at −80 °C. In total, 15–20 million cells were used to prepare nuclear extracts. Cell pellets were resuspended in 10 mM HEPES, pH 7.9, 2.5 mM MgCl_2_, 0.25 M sucrose, 0.1% NP40, 1 mM DTT, and 1× HALT protease inhibitors (ThermoFisher) and swelled for 10 min on ice. After centrifugation at 500*g*, nuclei were resuspended on ice in 25 mM HEPES, pH 7.9, 1.5 mM MgCl_2_, 700 mM NaCl, 0.5 mM DTT, 0.1 mM EDTA, 20% glycerol, 1 mM DTT, and 250 U benzonase, and incubated on ice for 10 min. Insoluble materials were pelleted by centrifugation at 18,000*g* at 4 °C for 10 min, and the supernatant (nuclear extracts) was stored at −80 °C. Protein concentration from supernatants was measured using the Pierce Coomassie Plus assay kit (Thermofisher).

For CTCF Western blot in *Extended* Fig. 2, 40 µg of nuclear extracts were loaded per lane. Samples were mixed with Laemmli buffer and 2.5% beta-mercaptoethanol, then loaded onto a Bolt 4–12% Bis–Tris Plus gel (ThermoFisher). Gels were wet-transferred onto PVDF membranes in transfer buffer (25 mM Tris-Base, 192 mM Glycine, and 10% Methanol) for 3 h at 80 V. Membranes were blocked for 2 h with Odyssey blocking buffer (Li-Cor cat. 927-40000) and subsequently incubated with primary antibody overnight at 4 °C (1:1000 anti-CTCF C-terminus Millipore 61311 and 1:2000 anti-TBP Abcam ab51841) in Odyssey blocking buffer. Membranes were washed three times in TBT–0.1% Tween, 5–10 min per wash, and were incubated with secondary antibodies at room temperature for 1 h (1:10,000 HRP-anti-rabbit Cell Sig #7074 and 1:10,000 HRP-anti-mouse Cell Sig #7076). Blots were washed 3 times for 5–10 min in TBS–0.1% Tween. CTCF blot used Amersham ECL Prime Western Blotting Detection Reagent (GE RPN2236) and TBP blot used Amersham ECL Western Blotting Detection Kit (GE RPN2108) for HRP activation. Blots were then exposed onto X-ray films for different exposure times.

### Co-immunoprecipitation

mESCs were dissociated, resuspended in culture medium, pelleted, washed in PBS, pelleted again, and kept at −80 °C. In total, 15–20 million cells were used for protein extraction. Pellets were thawed on ice and lysed in 10 mM Tris at pH 7.9 at 4 °C, 1.5 mM MgCl_2_, 10 mM KCl, 0.2% IGEPAL CA-630, and 1× Halt protease inhibitors (Thermofisher 78429) by incubating for 15 min on ice. Nuclei were pelleted by centrifugation at 2500*g* for 5 min at 4 °C and resuspended in 100 μL of 20 mM Tris at pH 7.9 at 4 °C, 25% glycerol, 400 mM NaCl, 1.5 mM MgCl_2_, 10 mM EDTA, 250 U benzonase, and 1× Halt protease inhibitors, and incubated on an orbital shaker for 60 min at 4 °C. Insoluble materials. Insoluble materials were pelleted by centrifugation at 18,000*g* at 4 °C for 10 min, and the supernatant (nuclear extracts) was diluted to 200 mM NaCl final by adding 100 μL of 20 mM Tris at pH 7.9 at 4 °C, 25% glycerol, 1.5 mM MgCl_2_, 10 mM EDTA, and 1× Halt protease inhibitors. Protein concentration from supernatants was measured using the Pierce Coomassie Plus assay kit (Thermofisher) and the concentration was adjusted to 1 mg/mL. In all, 3% input was set aside, and 500 μg of nuclear extracts were used for immunoprecipitation by adding 4 μg of anti-SA1 antibody (Abcam ab4457) and incubating for 3 h by rotation at 4 C. In the meantime, 25 μL Protein G beads (ThermoFisher) were washed twice with the 200 mM NaCL IP buffer and blocked for 1 h by adding 0.5% BSA final (Gemini 700-100 P). After blocking, beads were rinsed twice in the 200 mM NaCL IP buffer, resuspended in 25 μL of IP buffer, and added to the lysates for 1 h at 4 °C under rotation. Beads were then collected on a magnetic stand, rinsed three times with 200 mL of NaCL IP buffer, resuspended in 100 μL in 100 NaCL IP buffer containing 1× Laemli buffer (Biorad 1610737), and incubated at 95 °C for 5 min. Beads were then collected and discarded, and eluates were loaded equally on four separate 4–12% acrylamide gels (Biorad). Proteins were transferred onto PVDF membranes using the iBlot system (Thermofisher) Program 0 for 8 min. Membranes were incubated at least 30 min with Odyssey blocking buffer (Li-Cor) prior to antibody incubation overnight at 4 °C (anti-FLAG: Sigma-Millipore F3165; anti-SA1: Abcam 4457; anti-RAD21: Abcam 992; anti-SA2: Abcam 4463, using 10 μg of antibody in 10 mL of blocking buffer). Membranes were washed 3 times for 5 min in 1× PBS–0.1% Tween-20 at room temperature, incubated with secondary antibodies (Goat Anti-Rabbit 680RD and Donkey Anti-Mouse 800CW (Li-Cor), 1:10,000) in Odyssey blocking buffer with 0.1% Tween-20 and 0.01% SDS for 1 h at room temperature, washed 3 times, and analyzed on a Li-Cor imaging system. Panels were mounted using ImageJ preserving linearity.

### Reporting summary

Further information on research design is available in the Nature Research Reporting Summary linked to this article.

## Supplementary information

Supplementary Information

Reporting Summary

Description of Additional Supplementary Files

Supplementary Data 1

Supplementary Data 2

Supplementary Data 3

## Data Availability

The data that support this study are available from the corresponding authors upon reasonable request. Sequencing data presented in Figs. 2, 3, and 5 are available on Gene Expression Omnibus GEO GSE156868. We used the following publicly available datasets: GEO GSE98671, UniProtKB Q61164. Source data are provided with this paper

## Source data

Source Data

## Code availability

Scripts used to analyze imaging or genomic data are available upon request. Cooltools analysis software available at https://github.com/open2c/cooltools.
