# Supplementary material for: Molecular basis of CTCF binding polarity in genome folding
Source: Nat Commun. 2020 Nov 5;11:5612. doi: 10.1038/s41467-020-19283-x (PMC7645679; doi:10.1038/s41467-020-19283-x)
Supplement: Supplementary file 1 — Supplementary Information [file 41467_2020_19283_MOESM1_ESM.pdf]

Molecular basis of CTCF binding polarity in genome folding

Elphège P. Nora, Laura Caccianini, Geoffrey Fudenberg et al. 2020 Nature Communications

Supplementary information

Supplementary Fig. 1: Supporting information regarding RAD21 single molecule tracking in live cells

Supplementary Fig. 2: Supporting information regarding the CTCF complementation system

Supplementary Fig. 3: C(577-614) appears dispensable for connecting CTCF and cohesin functionally

Supplementary Fig. 4: Supporting data on the PDS5A-CTCF association

Supplementary Fig. 5: Hi-C analysis of CTCF truncations

Supplementary Fig. 6: Summary model for cohesin behavior after N- versus C-terminal encounters of CTCF

Supplementary Fig. 7: Rad21 is enriched at CTCF binding sites in Drosophila cells

Supplementary Fig. 8: Uncropped Western blots and flow-cytometry gating strategy.

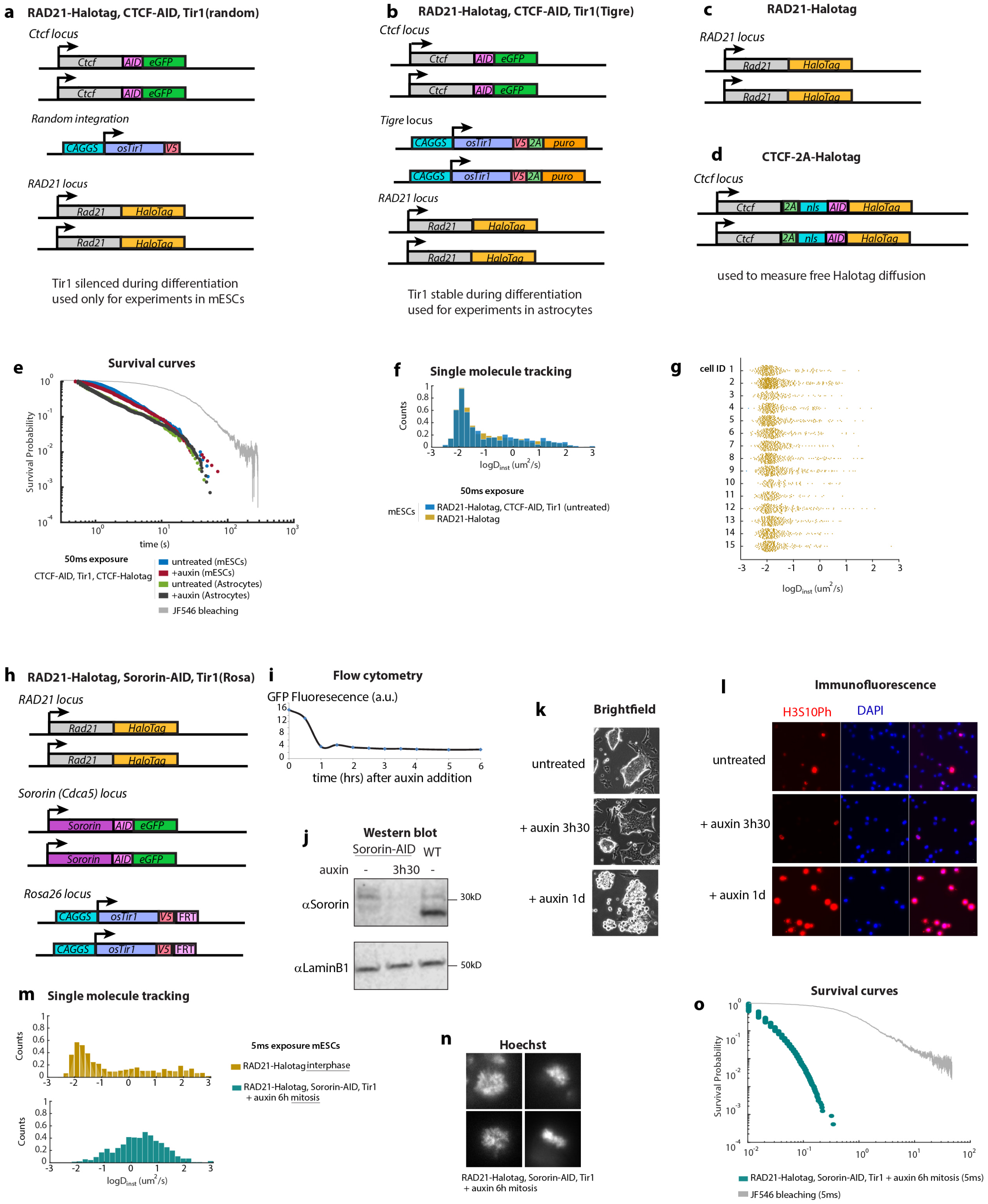

Supplementary Fig. 1: Supporting information regarding RAD21 single molecule tracking in live cells
a - **d**, detailed genotype of the Halotag cells used for live cell single molecule imaging.
e, survival curves estimated by combining and rescaling data acquired at 5, 50 and 500ms in CTCF-AID, RAD21-Halotag mESCs. Survival Probability computed from data acquired with continuous imaging at

50ms exposure. Statistics: mESC untreated: 507 trajectories; mESC +auxin: 731 trajectories; astrocytes untreated: 628 trajectories; astrocytes +auxin: 1090 trajectories N = 15 cells per condition.
f, Similar distribution of RAD21-Halotag diffusion coefficient in cells with or without CTCF-AID
g, RAD21-Halotag single mESCs display similar Diffusion coefficients
h, detailed genotype of the SORORIN-AID-eGFP, Tir1, RAD21-Halotag cells
i, depletion kinetics of SORORIN-AID after auxin addition using flow cytometry for GFP in mESCs
j, Western blot indicating destabilization of SORORIN after addition of the AID tag and complete disappearance after auxin treatment. Experiment performed once.
k, brightfield imaging of live SORORIN-AID-eGFP mESCs illustrating the accumulation of round refringent mitotic cells after incubation with auxin for 1 day (doubling time of parental cells = 12-14hrs). Mitotic block was systematically observed across experiments.
l, H3S10 immunofluorescence confirming the accumulation of mitotic cells after auxin treatment of SORORIN-AID cells. Experiment performed once.
m, rapid diffusion coefficients observed mitotic cells
n, examples of the mitotic figures in cells analyzed
o, RAD21-Halotag binding events detected in mitotic cells have very short survival time.
Source data are provided as a Source Data file

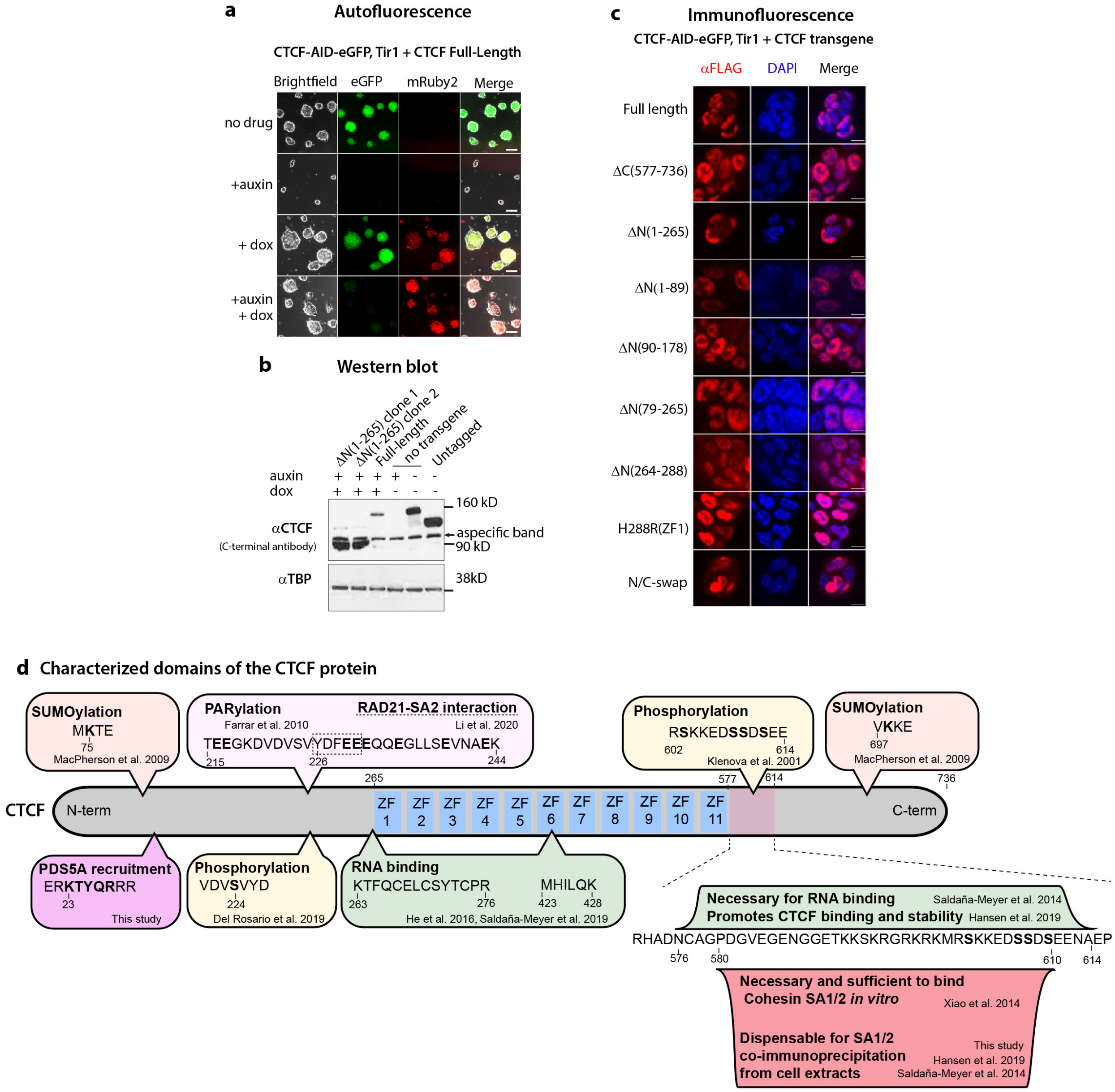

Supplementary Fig. 2: Supporting information regarding the CTCF complementation system

a, Brightfield images of live complemented mESCs after 4 days of treatment. scale bar = 100μm.

b, Western blot with a C-terminal antibody after 2 days of treatment (Milipore 07-729) confirming slightly higher expression of the CTCF Δ(1-265)

c, all CTCF truncation analyzed displayed nuclear localization using Immunofluorescence against the FLAG tag after 2 days of treatment.

d, schematic depiction of known functional and post-translationally modified sites in CTCF. Amino-acid numbers refer to the mouse protein. When information was only available in humans, orthologous amino-acids are reported on the figure.

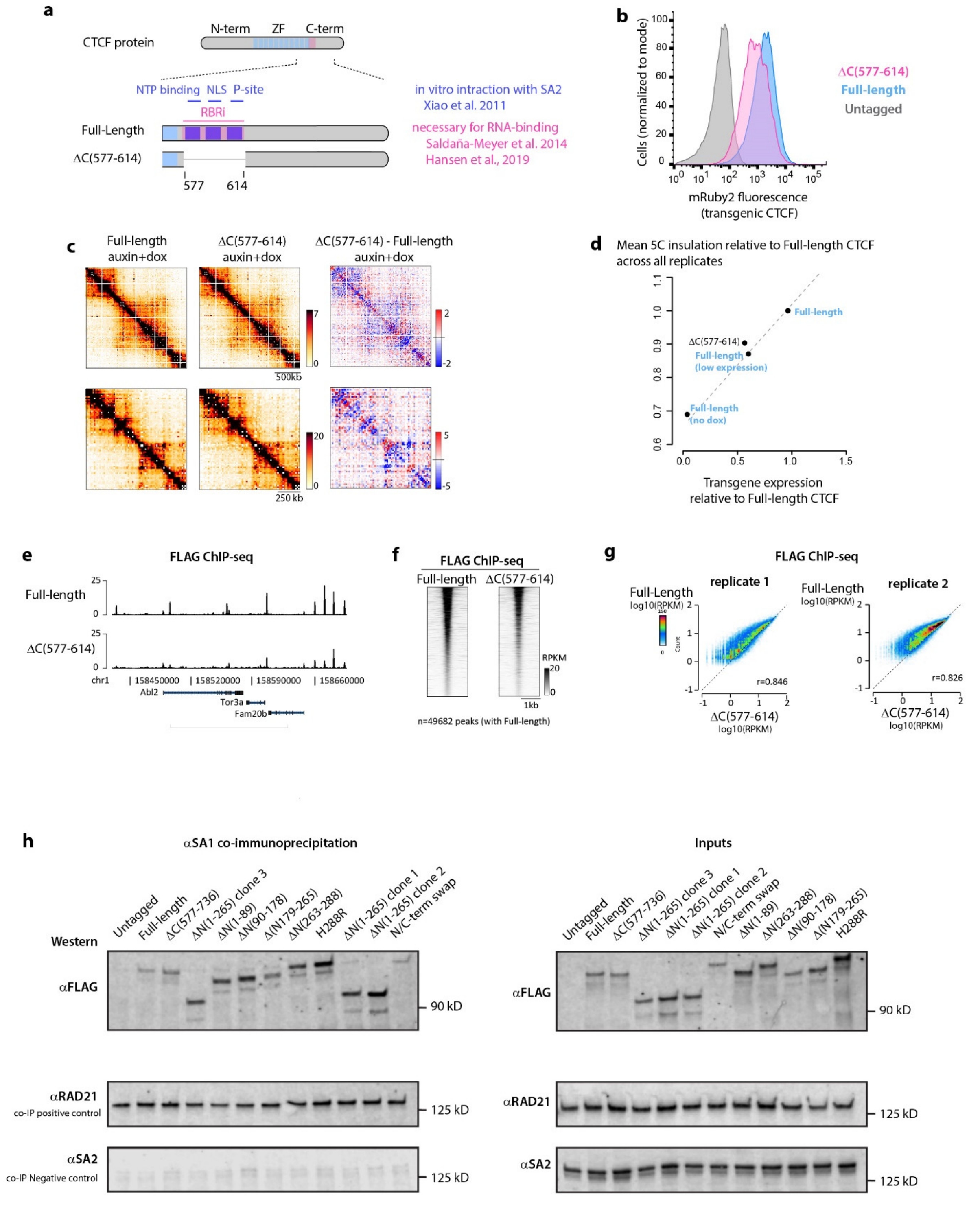

Supplementary Fig. 3: C(577-614) appears dispensable for connecting CTCF and cohesin functionally

a, schematic depiction of the position of the Xiao et al. 2011 cohesin SA interaction domain and C-terminal RNA binding region (RBR_i).

b, flow cytometry illustrating lower expression level of the ΔC(577-614) transgenic CTCF

c, 5C snapshots in ΔC(577-614) binned at 15kb.
d, excerpt of Figure 2b displaying only the ΔC(577-614) mutant.
e-g, ΔC(577-614) displays lower overall binding to DNA by ChIP-seq than control full-length CTCF with higher transgene expression
h, SA1 immunoprecipitation followed by FLAG, RAD21 (positive control) or SA2 (negative control) Western blot in various CTCF truncations, after a 4-day treatment of auxin + doxycycline. Experiment performed once with all mutants.

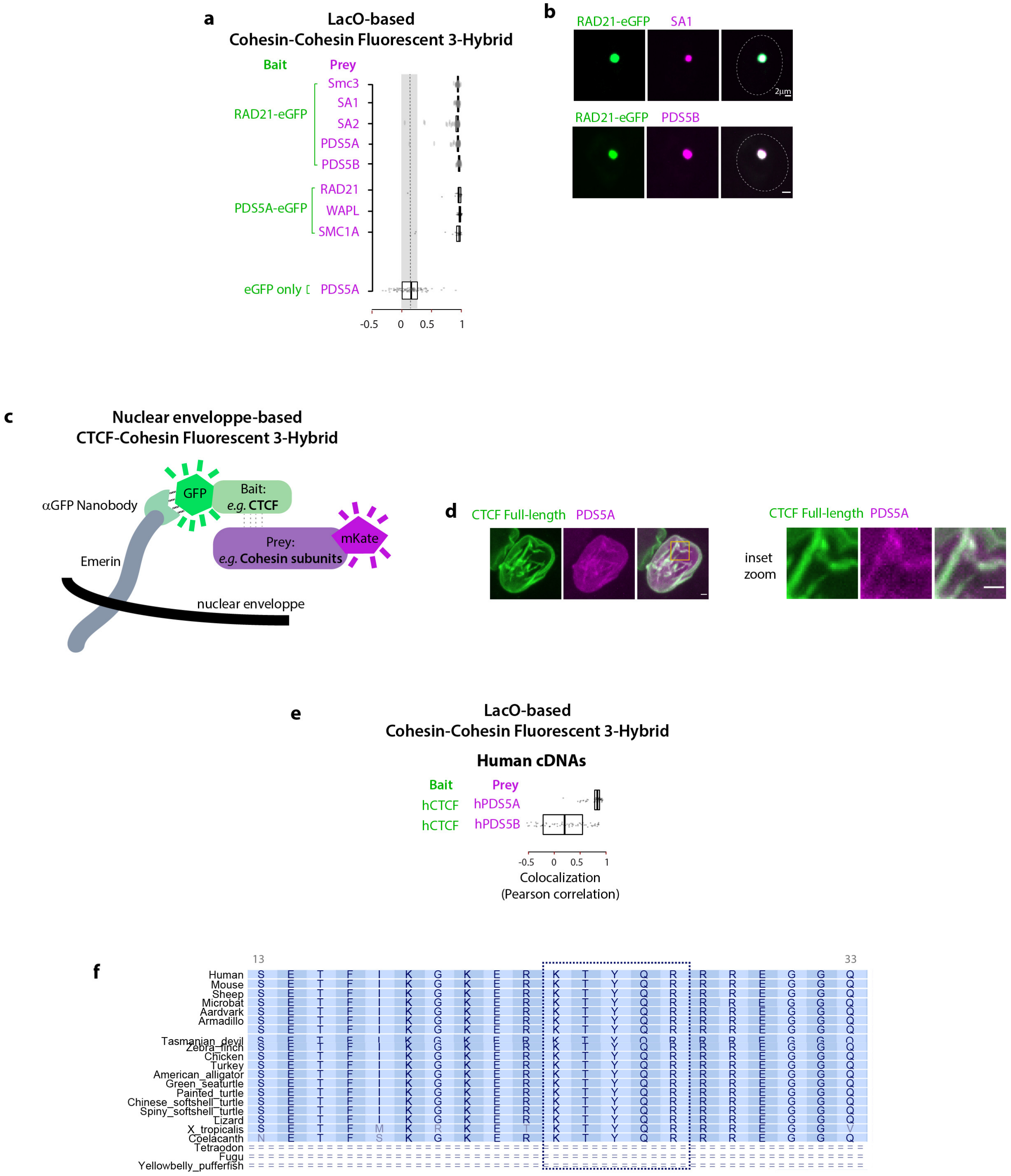

Supplementary Fig. 4: Supporting data on the PDS5A-CTCF association

a, Positive controls for the Fluorescent three-hybrid, demonstrating that all the cohesin-mKate fusions can be recruited by known interactors (other cohesin subunits). Note that colocalization scores between cohesin subunits are significantly higher than for CTCF-eGFP and PDS5A-mRuby as shown in figure 3.

b, examples of fixed cell images.

c, schematic of the F3H at the nuclear envelope using Emerin as a tether.

d, examples of fixed cell images illustrating that CTCF can recruit PDS5A even when tethered to the Emerin protein instead of the LacO DNA. Experiment performed twice with similar observation.
e, F3H using human cDNAs showing confirming that CTCF recruits more efficiently PDS5A than PDS5B. Boxplots indicate first and third quartile and median.
f, Evolutionary alignment of mouse CTCF 13-33 protein sequence. Box: KTYQR motif necessary for PDS5A recruitment and homologous to the PDS5-interacting domain of WAPL, SORORIN and HASPIN.
Source data are provided as a Source Data file

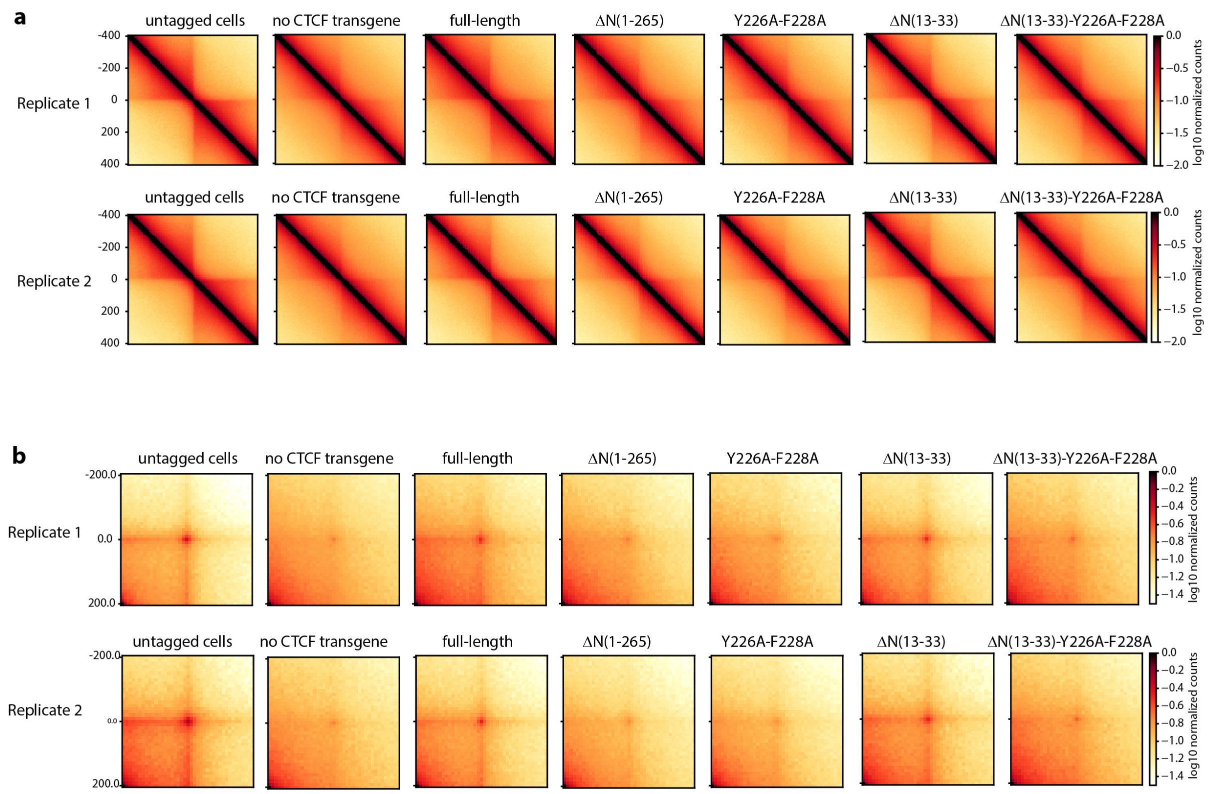

Supplementary Fig. 5: Hi-C analysis of CTCF truncations

a, meta-TAD analysis for each replicate separately as presented in Fig.5b

b, meta-peak analysis for each replicate separately as presented in Fig.5c

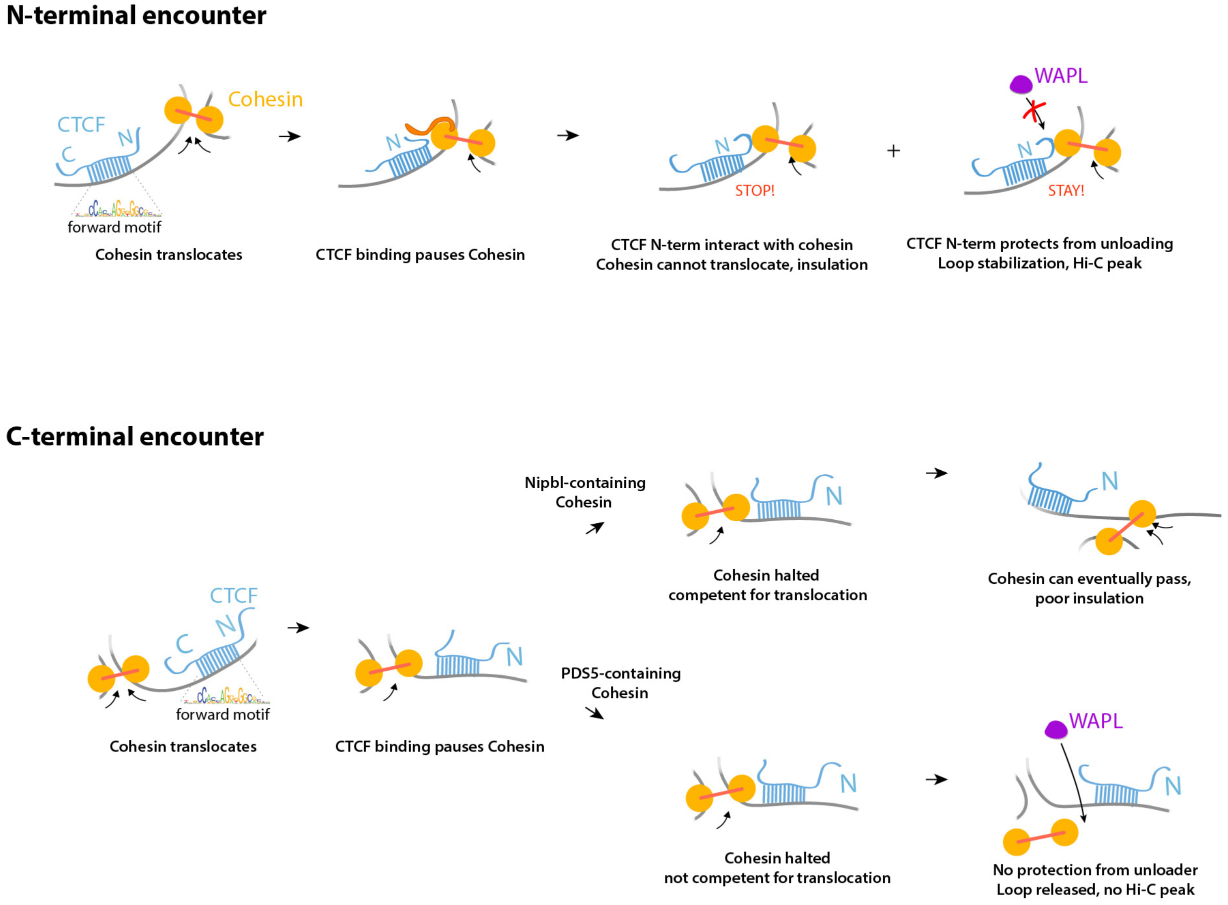

Supplementary Fig. 6: Summary model for cohesin behavior after N- versus C-terminal encounters of CTCF

N-terminal encounter: Upon deleting the N-terminus of CTCF, cohesin occupancy is diminished but still detectable, indicating that cohesin still pauses upon encountering bound CTCF sites. Loss of cohesin occupancy can be attributed to both decreased ability of truncated CTCF to block cohesin (leading to insulation defects), as well as decreased ability of truncated CTCF to protect halted cohesin from unloading (leading to loss of the DNA loop). **C-terminal encounter:** A similar sequence of events occurs when cohesin encounters normal CTCF from the C-terminal side or N-terminus truncated CTCF from either side: CTCF binding pauses cohesin, and resolution of that pause would depend on whether NIPBL or PDS5 remains bound to cohesin, given these two co-factors bind exclusively to cohesin[36] but in a dynamic fashion (at least for NIPBL)[54]. NIPBL-containing complexes remain competent for translocation and would eventually pass, with no or poor insulation detected by 5C. PDS5-containing complexes, although not competent for translocation, can be recognized by Wapl for unloading and would therefore ultimately release the DNA loop since the CTCF N-terminus cannot protect them.

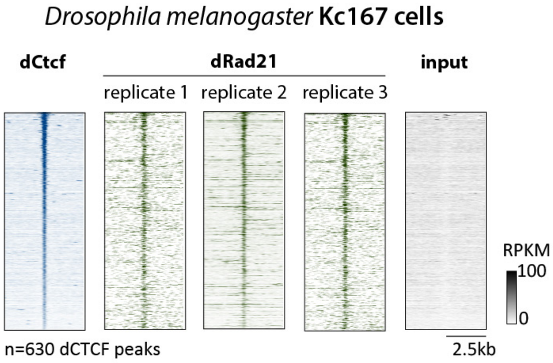

Supplementary Fig. 7: Rad21 is enriched at CTCF binding sites in Drosophila cells

Density plots of CTCF and Rad21 ChIP-seq signal centered at CTCF peaks in Drosophila Kc167 cells[45].

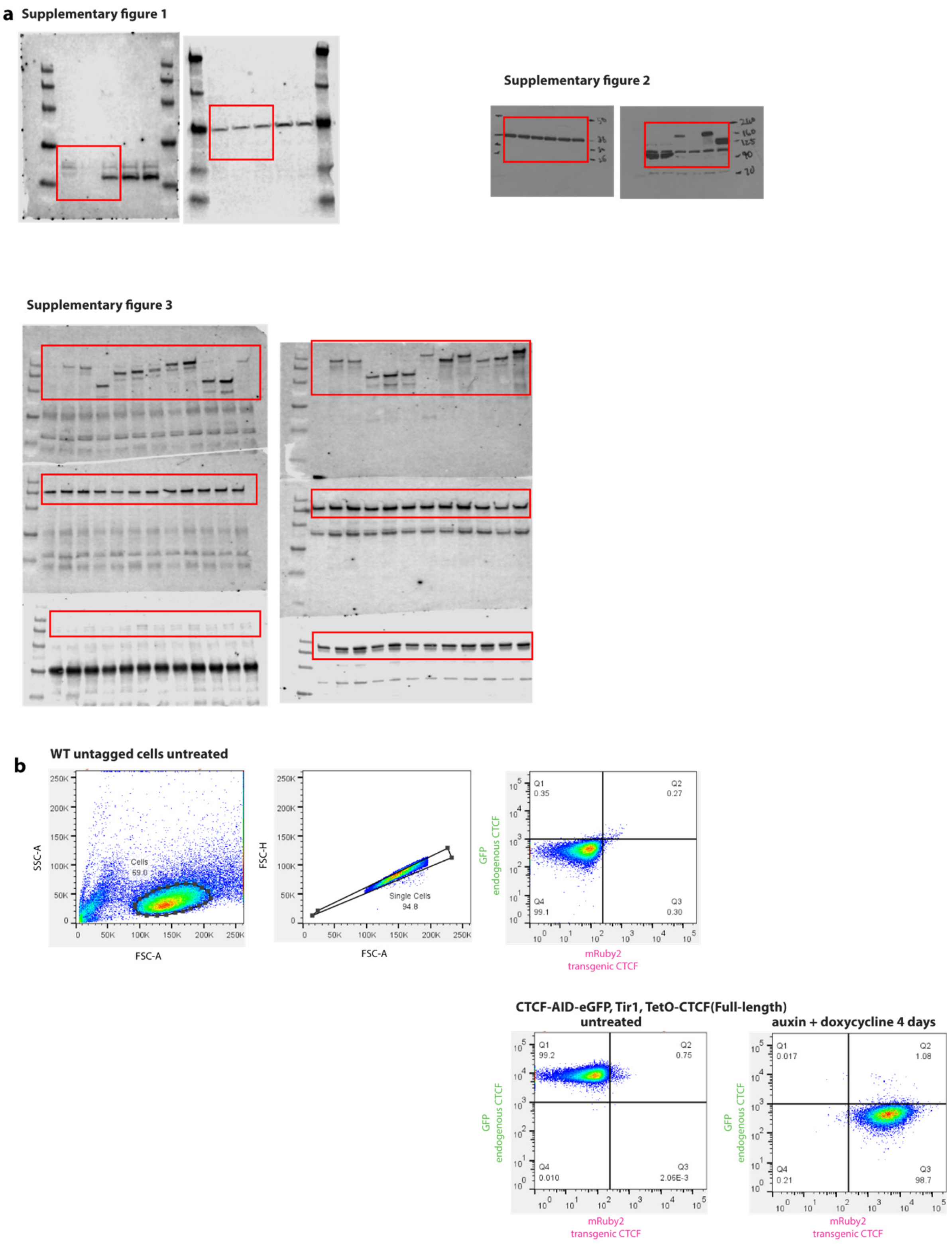

Supplementary Fig. 8:

a, Uncropped Western blots

b, Flow-cytometry gating strategy
