# Supplementary material for: Molecular basis of CTCF binding polarity in genome folding
Source: Nat Commun. 2020 Nov 5;11:5612. doi: 10.1038/s41467-020-19283-x (PMC7645679; doi:10.1038/s41467-020-19283-x)
Supplement: Supplementary file 2 — Reporting Summary [file 41467_2020_19283_MOESM2_ESM.pdf]

nature research

Corresponding author(s): Benoit Bruneau, Elphege Nora

Last updated by author(s): Aug 25, 2020

Reporting Summary

Nature Research wishes to improve the reproducibility of the work that we publish. This form provides structure for consistency and transparency in reporting. For further information on Nature Research policies, see our Editorial Policies and the Editorial Policy Checklist.

Statistics

For all statistical analyses, confirm that the following items are present in the figure legend, table legend, main text, or Methods section.

n/a	Confirmed	
☐	☒	The exact sample size (*n*) for each experimental group/condition, given as a discrete number and unit of measurement
☐	☒	A statement on whether measurements were taken from distinct samples or whether the same sample was measured repeatedly
☐	☒	The statistical test(s) used AND whether they are one- or two-sided<br>*Only common tests should be described solely by name; describe more complex techniques in the Methods section.*
☒	☐	A description of all covariates tested
☒	☐	A description of any assumptions or corrections, such as tests of normality and adjustment for multiple comparisons
☐	☒	A full description of the statistical parameters including central tendency (e.g. means) or other basic estimates (e.g. regression coefficient) AND variation (e.g. standard deviation) or associated estimates of uncertainty (e.g. confidence intervals)
☒	☐	For null hypothesis testing, the test statistic (e.g. *F*, *t*, *r*) with confidence intervals, effect sizes, degrees of freedom and *P* value noted<br>*Give P values as exact values whenever suitable.*
☒	☐	For Bayesian analysis, information on the choice of priors and Markov chain Monte Carlo settings
☒	☐	For hierarchical and complex designs, identification of the appropriate level for tests and full reporting of outcomes
☐	☒	Estimates of effect sizes (e.g. Cohen's *d*, Pearson's *r*), indicating how they were calculated

Our web collection on statistics for biologists contains articles on many of the points above.

Software and code

Policy information about availability of computer code

Data collection	Sequencing data was collected on an illumina Next seq instrument with built in proprietary softwares.
Data analysis	Analytical softwares used in this paper are cited in the Online Methods section.

For manuscripts utilizing custom algorithms or software that are central to the research but not yet described in published literature, software must be made available to editors and reviewers. We strongly encourage code deposition in a community repository (e.g. GitHub). See the Nature Research guidelines for submitting code & software for further information.

Data

Policy information about availability of data

All manuscripts must include a data availability statement. This statement should provide the following information, where applicable:

- Accession codes, unique identifiers, or web links for publicly available datasets
- A list of figures that have associated raw data
- A description of any restrictions on data availability

All sequencing data is publicly available on GEO under accession GSE156868
We used the following publically available datasets: GEO GSE98671 (https://www.ncbi.nlm.nih.gov/geo/query/acc.cgi?acc=GSE98671), UniProtKB Q61164 (https://www.uniprot.org/uniprot/Q61164)

Field-specific reporting

Please select the one below that is the best fit for your research. If you are not sure, read the appropriate sections before making your selection.

☒ Life sciences ☐ Behavioural & social sciences ☐ Ecological, evolutionary & environmental sciences

For a reference copy of the document with all sections, see nature.com/documents/nr-reporting-summary-flat.pdf

Life sciences study design

All studies must disclose on these points even when the disclosure is negative.

Sample size	sample size was not predetermined. At least two bioloigcal replicates for each experiments, and 5C and Hi-C in particular was chosen based on common standards of the field.
Data exclusions	no data was excluded
Replication	all experiments were performed in at least two biological replicates. all attempts were successful.
Randomization	no randomization was performed
Blinding	investigators were not blinded

Reporting for specific materials, systems and methods

We require information from authors about some types of materials, experimental systems and methods used in many studies. Here, indicate whether each material, system or method listed is relevant to your study. If you are not sure if a list item applies to your research, read the appropriate section before selecting a response.

Materials & experimental systems

n/a	Involved in the study	
☐	☒	Antibodies
☐	☒	Eukaryotic cell lines
☒	☐	Palaeontology and archaeology
☒	☐	Animals and other organisms
☒	☐	Human research participants
☒	☐	Clinical data
☒	☐	Dual use research of concern

Methods

n/a	Involved in the study	
☐	☒	ChIP-seq
☐	☒	Flow cytometry
☒	☐	MRI-based neuroimaging

Antibodies

Antibodies used

anti-FLAG: Sigma-Millipore F3165 and F1804
AlexaFluor594 Goat anti-Mouse IgG Invitrogen A--11005
Anti-H3S10Ph, rabbit polyclonal Millipore 05-636
anti-TBP Abcam ab51841
anti-CTCF C-terminus Millipore 61311
HRP-anti-mouse Cell Sig #7076
HRP-anti-rabbit Cell Sig #7074
anti-SA1 antibody (Abcam ab4457)
anti-SA2: Abcam 4463
Goat Anti-Rabbit 680RD Li-Cor
Donkey Anti-Mouse 800CW Li-Cor
RAD21 abcam ab992
anti-H2Av spike in antibody Active motif 61686

Validation

antibodies validation on manufacturer's website.

validation of primary antibodies:
The use of the anti-FLAG: Sigma-Millipore F3165 and F1804 for ChIP-seq was validate by observing that the FLAG-CTCF ChIP-seq profile was highly similar to published CTCF ChIP-seq
AlexaFluor594 Goat anti-Mouse IgG Invitrogen A--11005
The use of the Anti-H3S10Ph, rabbit polyclonal Millipore 05-636 for immunofluorescence was validated by observing expected

nuclear staining pattern in WT cells
The use of the anti-TBP Abcam ab51841 for western blot was validated by observing expected molecular weight on western blots
The use of the anti-CTCF C-terminus Millipore 61311 for western blot was validated by observing expected molecular weight on western blots

The use of the anti-SA1 antibody (Abcam ab4457) or western blot was validated by observing expected molecular weight on western blots
The use of the anti-SA2: Abcam 4463 or western blot was validated by observing expected molecular weight on western blots
The use of the RAD21 abcam ab992 for ChIP-seq was validated by observing a largely overlapping profile with that of published CTCF ChIP-seq
The use of the anti-H2Av spike in antibody Active motif 61686for ChIP-seq was validated by observing a largely overlapping profile with that of published H2av ChIP-seq on the drosophila genome (spike in chromatin)

Eukaryotic cell lines

Policy information about cell lines

Cell line source(s)	E14 mouse ES cells and derived edited clones as described in the methods section<br>BHK LacO clones#2 cells initially described in Tsukamoto et al 2000 Nat Cell Biol and kindly provided by Dr. Pierre-Antoine Defossez.<br>S2 Schneider's Drosophila Line 2 [D. Mel. (2), SL2] ATCC CRL-1963
Authentication	E14 cells were not authenticated for this study.<br>BHK cells were authenticated by the detection of the LacO array as described in the method sections<br>The S2 cells were obtained directly form ATCC but not authenticated for this study
Mycoplasma contamination	cells were tested quarterly for mycoplasma and were negative
Commonly misidentified lines (See ICLAC register)	no commonly misidentified cell lines were used in the study.

ChIP-seq

Data deposition

☒ Confirm that both raw and final processed data have been deposited in a public database such as GEO.

☒ Confirm that you have deposited or provided access to graph files (e.g. BED files) for the called peaks.

Data access links<br>*May remain private before publication.*	GEO GSE156868
Files in database submission	see dataset listed in supplementary table
Genome browser session (e.g. UCSC)	no longer applicable

Methodology

Replicates	see dataset listed in supplementary table
Sequencing depth	see dataset listed in supplementary table
Antibodies	anti-FLAG: Sigma-Millipore F3165 and F1804<br>RAD21 abcam ab992<br>anti-H2Av spike in antibody Active motif 61686<br>lot number not available
Peak calling parameters	peaks were called using matched inputs as identified in the supplementary Table
Data quality	NA. The RAD21 ChIP-seq analysis involved computing the number of ChIP-seq counts over published CTCF ChIP-seq peaks. We did not compute RAD21 ChIP-seq peaks.
Software	Fastq files were trimmed using the fastq-mcf program, aligned to the mm9 reference genome with bowtie2 (Langmead and Salzberg, 2012). Reads with a mapq score of 30 or greater were retained, using Samtools. Chip-seq peaks were called on each replicate individually using all available reads. For peak calling we followed the guidelines described in (Thomas et al., 2016). For CTCF, which display focal enrichment, we used the Genome-wide Event finding and Motif discovery (GEM) method (Guo et al., 2012). The consensus peak list was obtained by retaining peaks that overlapped for at least 1bp between biological replicates.

Flow Cytometry

Plots

Confirm that:

- [x] The axis labels state the marker and fluorochrome used (e.g. CD4-FITC).
- [x] The axis scales are clearly visible. Include numbers along axes only for bottom left plot of group (a 'group' is an analysis of identical markers).
- [x] All plots are contour plots with outliers or pseudocolor plots.
- [ ] A numerical value for number of cells or percentage (with statistics) is provided.

Methodology

Sample preparation	mESCs were dissociated with TryplE, resuspended in culture medium, spun, and resuspended in 4% FBS-PBS before live flow cytometry
Instrument	MACSQuant instrument (Miltenyibiotec).
Software	Flowjo sowftware.
Cell population abundance	no sorting was performed
Gating strategy	gating strategy is outlined in detailed in the supplementary figure.

- [ ] Tick this box to confirm that a figure exemplifying the gating strategy is provided in the Supplementary Information.
