# Supplementary material for: Molecular basis of CTCF binding polarity in genome folding
Source: Nat Commun. 2020 Nov 5;11:5612. doi: 10.1038/s41467-020-19283-x (PMC7645679; doi:10.1038/s41467-020-19283-x)
Supplement: Supplementary file 3 — Description of Additional Supplementary Files [file 41467_2020_19283_MOESM3_ESM.pdf]

Description of Additional Supplementary Files

File name: Supplementary Data 1

Description: Cell Lines and Vectors

File name: Supplementary Data 2

Description: Sequencing statistics

File name: Supplementary Data 3

Description: Single molecule imaging statistics
